# The Efficacy and Safety of Chinese Herbal Medicine Xianling Gubao Capsule Combined With Alendronate in the Treatment of Primary Osteoporosis: A Systematic Review and Meta-Analysis of 20 Randomized Controlled Trials

**DOI:** 10.3389/fphar.2021.695832

**Published:** 2021-07-16

**Authors:** Jiaru Chen, Junju Zheng, Mangmang Chen, Shenglei Lin, Zhou Lin

**Affiliations:** Department of Orthopaedic Surgery, Wenzhou Central Hospital, Wenzhou, China

**Keywords:** Chinese herbal formula, osteoporosis, xianling gubao capsule, alendronate, systematic review and meta-analysis

## Abstract

**Objective:** Herein, we purposed to evaluate the efficacy along with the safety of Xianling Gubao capsule (XLGB) combined with alendronate (ALE) for primary osteoporosis (POP) from the current literature.

**Materials and Methods:** We carried out a search for electronic literature in the PubMed, Chinese National Knowledge Infrastructure, EMBASE, Wanfang Web of Science, Chinese Biomedical Literature Database, Cochrane Library, as well as Chinese VIP databases targeting articles published from inception to December 2020. Only randomized controlled trials (RCTs) were enrolled into the study. Alkaline phosphatase (ALP), visual analogue scale (VAS), serum phosphorus (S-P), bone gla protein (BGP), serum calcium (S-Ca) and bone mineral density (BMD) were the primary outcome variable. The total clinical effective rate along with the adverse drug reaction (ADR) were the secondary outcome variables. The meta-analysis was conducted using RevMan 5.3 and STATA 12.0. GRADE pro3.6.1 software was used for the assessment of evidence quality.

**Results:** Overall, 20 RCTs focusing on 1911 patients were enrolled into the study. Our meta-analysis demonstrated that XLGB combined with ALE remarkably increased BMD (*p* < 0.001), BGP (*p* < 0.001), S-Ca (*p* < 0.001), S-P (*p* < 0.001) and effective rate (*p* < 0.001) than ALE alone in patients with POP. Moreover, ALP (*p* < 0.001) and VAS (*p* < 0.001) were overtly by decreased XLGB. However, XLGB combined with ALE would not markedly increase the rate of ADR in contrast with ALE alone (*p* = 0.499).

**Conclusion:** The results of our study demonstrated that XLGB is a potential candidate for OP treatment. We recommend that rigorous, as well as high-quality trials involving large samples sizes should be conducted to confirm our findings.

## Introduction

OP is a progressive skeletal condition which is chronic and manifests through reduced bone mass along with microarchitectural deterioration, resulting in elevated chances of fracture (Cosman, et al., 2014). The condition can elevate bone fragility, consequently increasing bone fracture risk particularly in the spine, hip, as well as wrist (Rachner et al., 2011). Reports show that about 9.9 million of the Americans suffer from osteoporosis, with an additional 43.1 million experiencing low BMD (Wright et al., 2014). OP prevalence in China was 14.94% before 2008 and increased to 27.96% from 2012 to 2015, with the rate being higher in females relative to males (Chen et al., 2016). Analogously, in the United States, 16% of men and 29.9% of women aged more than 50 years have OP on the basis of the diagnostic criteria of the NBHA (National Bone Health Alliance) (Wright et al., 2017). OP is categorized into two classes, namely secondary and POP. Secondary OP is caused by any disease and/or drug, which affects the bone remodeling, as well as other known causes (Si et al., 2015). On the other hand, primary OP consists of post-menopause OP, senile OP, and idiopathic OP. Post-menopause OP often develops at five to ten years post menopause, while senile OP primarily develops in older individuals with more than 70 years. Idiopathic OP primarily develops in adolescents, however, its etiology remains unclear. OP has been attributed to decreased quality of life, increased risks of death along with elevated burden on health systems economically (Orive et al., 2015). Therefore, the management of patients with osteopenia or OP is extremely urgent.

In the recent three decades, there have been remarkable advancements in the therapy approaches of OP (Bolland et al., 2010). The current treatment agents have been designed to maintain, as well as increase bone mass, at the same minimize chances of bone fractures. The OP treatment medications are classified into two groups, namely the anabolic drugs and the antiresorptive agents. The antiresorptive drugs consist of estrogen agonist/antagonists (EAAs), bisphosphonates, estrogens, as well as calcitonin. The anabolic agents include teriparatides (Ettinger, 2003; Lewiecki, 2010; Tu et al., 2018). Bisphosphonates are extensively employed to avert or treat OP through the induction of the apoptosis of osteoclasts and repressing resorption of bone tissue (Rogers, 2003). Of note, ALE is the most prescribed bisphosphonates, and has been documented to be effective and well-tolerated in preventing and treating OP (Adachi et al., 2001). A previous research documented that ALE reduces chances of vertebral fractures in men (Sawka et al., 2005). Besides, Okada et al. demonstrated that ALE provides protection in pre-menopausal women against fractures linked to high-dose glucocorticoid treatment and bone loss (Okada et al., 2008). However, despite the availability of numerous anti-OP medications with diverse pharmacological properties, as well as fixed-dose combination therapy, the targeted therapeutic effect is not attained in significant numbers of individuals with OP, and the mitigation of OP fracture has remained suboptimal (Burch et al., 2014; Ishtiaq et al., 2015).

Consequently, it is highly required to seek out newer therapeutic options or agents to treat OP. Recently, the growing utilization of complementary and alternative medicine, consisting of Chinese herbal medicine in treating OP has attracted extensive attention (Zhang et al., 2016; Li et al., 2017; Wang et al., 2018). XLGB is a frequently used Chinese herbal formula, has been widely used in OP, osteoarthritis, aseptic necrosis of head of femur and osteoporotic fracture in China (Xing et al., 2013). Its composition includes Epimedium, Dipsacus, Psoralen, Rehmannia glutinosa, Salvia miltiorrhiza, and Anemarrhena. It has the functions of nourishing liver and kidney, promoting blood circulation and clearing collaterals, strengthening muscles, and bones (Ni et al., 2011). A previous study has demonstrated that the component of XLGB can prevent ovariectomized (OVX)-triggered bone loss verified with biomarkers of bone mass, bone turnover, bone microarchitecture along with bone strength in mice and also effectively promotes osteoblast-like UMR 106 cell proliferation, as well as mineralization, *in vitro* (Wang et al., 2015). Besides, a multicenter RCT illustrated that treatment with conventional dose of XLGB for over one year is safe and remarkably increases the lumbar spine BMD at six months in postmenopausal women (Zhu et al., 2012). The main component of XLGB epimedium exerted a favorable influence by mitigating the loss of bone tissue in late post-menopausal women without leading to an evident hyperplasia influence on the endometrium (Zhang et al., 2007). More importantly, the authoritative academic institutions in China issued the latest guidelines on OP clinical diagnosis and treatment recommending the use of XLGB (Xie, et al., 2012; Osteoporosis and Bone Mineral Disease Branch of Chinese Medical Association, 2017; Osteoporosis Branch of Chinese Society of Gerontology and Gerontology, 2019). Numerous appropriate clinical trials on the safety and effect of XLGB combined with ALE have been conducted (Ye et al., 2018; Zhang et al., 2020); however, no systematic reviews or meta-analysis has been conducted on the efficacy, as well as the safety of XLGB combined with ALE focused on treating POP. Therefore, there is no research evidence on the use of a combination regimen of XLGB with ALE to treat POP. Thus, it is critical to conduct a meta-analysis on the safety and efficacy of XLGB add-on treatment in individuals with POP. Recently, growing number of high-quality RCTs have documented on the safety and effectiveness of a regimen of XLGB and ALE combination for treating POP. Hence, herein, we carried out a large sample-sized systematic review and meta-analysis involving high-quality RCTs to evaluate the effect and safety of XLGB combined ALE in treating POP. We aimed to provide research evidence for clinical practice.

## Methods

A systematic review and meta-analysis was carried out on the basis of the Preferred Reporting Items for Systematic Reviews and Meta-Analyses (PRISMA) (Moher et al., 2009), as well as Assessing the methodological quality of systematic reviews (AMSTAR) guidelines. This study has been registered in Research Registry (https://www.researchregistry.com/), registration number is reviewregistry1056. We have not collected any primary personal data; therefore, ethical approval was not need.

### Database and Search Strategies

We performed electronic exploration in eight repositories, namely, PubMed, Chinese Biomedical Literature Database, Web of Science, Wanfang Database, EMBASE, Chinese National Knowledge Infrastructure, Cochrane Library, and Chinese VIP Database, from their respective establishment to December 2020. Additionally, we performed manual searches of extra relevant literature in the references of previously published systematic reviews. Moreover, the literature search was not limited to any language of publication. The search strategy in the English databases included: ([“Xianling Gubao Capsule”] OR [“Xianlinggubao Capsule”] OR [“Xianling Gubao”] OR [“Xianlinggubao”]) AND ([“alendronate”] OR [“alendronic acid”]) AND ([“osteoporosis”] OR [“primary osteoporosis”] OR [“postmenopausal osteoporosis”] OR [“senile osteoporosis”] OR [“age-related osteoporosis”]) AND ([“random control trials”] OR [“RCT”]). The free text terms used to search the Chinese databases, included ([“Xianling Gubao”]) and ([“a lun linsuan (which means alendronate in Chinese)”] and ([“gu zhi su song (which means osteoporosis in Chinese)”] or [“yuan fa xing gu zhi su song (which means primary osteoporosis in Chinese)”] or [“jue jing hou gu zhi su song (which means postmenopausal osteoporosis in Chinese)”] or [“lao nian xing gu zhi su song (which means senile osteoporosis in Chinese)”] or [“nian ling xiang guan xing gu zhi su song (which means age-related osteoporosis in Chinese)”]) and “sui ji dui zhao shi yan (which means RCT in Chinese)”.

### Eligibility Criteria

#### Types of Studies

We only included RCTs that investigated the safety and efficacy of XLGB combined with ALE for OP without any limitation regarding the status of publication or language. If we found a relevant study with three arms of treatment, only data for the arm (s) entailing XLGB and the control arm (s) were extracted. We excluded quasi-randomized trials, including studies where the subjects were allocated based on the date of birth, as well as the order of admission number.

#### Types of Participants

We enrolled subjects diagnosed with primary OP (Kanis et al., 1994; Society, 2000) without regard to disease course and severity age, and gender. Additionally, we applied other diagnostic criteria with comparable definitions. Postmenopausal OP and senile OP are considered POP.

#### Types of Interventions

The examined treatment intervention was XLGB combined with ALE, notwithstanding the dosage, duration, administration route, administrated approaches or the administration period of therapy. The comparator was treated with ALE alone.

#### Types of Outcome Measures

The primary outcome parameters included: 1) ALP 2) BGP 3) S-Ca 4) S-P 5) VAS 6) BMD, including BMD-lumbar spine, BMD-femoral neck and BMD-Ward’s area). ADR and the total clinical effective rate were the secondary variables.

### Exclusion Criteria

1) Non-randomized or quasi-RCT;
2) RCTs with participants who were not diagnosed with OP;
3) combined XLGB with other drugs;
4) Meeting abstracts, reviews, as well as animal experiments;
5) Duplicate publications, as well as RCTs with insufficient data.

### Literature Selection

The PRISMA flow diagram was utilized in selecting the included studies. We imported the literature results into the Endnote X7 software. Two independent authors evaluated the prospective eligible articles by first screening the titles and abstracts to remove duplications and irrelevant studies or the RCTs outside the inclusion criteria. After that, we downloaded the full-texts of the remaining prospective studies and then reviewed them. Any discord between the first two authors was deliberated with a third independent investigator.

### Data Extraction

Two independent reviewers retrieved the data, and then a third independent reviewer examined the uniformity. A standard form was utilized consisting of the retrieved items, including the general information of the study: the name(s) of the author (s), date of publication, criteria used in diagnosis, study design, age of participants, sample number, intervention method involving XLGB and ALE, course of treatment, and duration of disease. Regarding continuous outcomes, we retrieved the mean, number of participants, as well as the standard deviation (SD) in the respective study. Regarding dichotomous outcomes, the overall number as well as the number of the events of the XLGB group along with control group were retrieved. Where possible, we re-computed the data in other forms to allow for pooled analysis. Discords between these two reviewers, if any, were solved via discussions. Where necessary, we reached out to the corresponding authors of the included studies to provide us with the missing data or any extra information.

### Assessment of the Quality of Enrolled Studies

Two independent authors evaluated the quality of methodology as well as the risk of bias of the enrolled RCT studies utilizing the Cochrane collaboration tool (Higgins and Green, 2011). This Cochrane tool evaluates following parameters: randomization, allocation concealment, blinding of subjects, outcome evaluation blinding, selective outcome reporting, incomplete outcome data, as well as other bias, for each item, categorizes studies into unclear, low, or high risk of bias.

### Evidence Quality Assessment

The GRADE criteria was employed to assess the quality of the evidence (Balshem, et al., 2011; Guyatt, et al., 2011). The quality of evidence of the meta-analysis outcomes categorized into either very low, low, moderate, or high. Initially, the RCT outcomes were ranked as high-quality evidence. Each outcome’s quality was de-graded because of the following factors: risk of bias, imprecision, inconsistency, publication bias, and indirectness. GRADE pro3.6.1 software was employed to perform data analysis, as well as synthesis.

### Statistical Analysis

All the retrieved data herein was analyzed in the STATA software (V.12.0; StataCorp, College Station, TX) for meta-analysis. If high statistical heterogeneity (*p* < 0.05 or *I* ^2^ > 50%) was found, a random-effects model was applied, further subgroup analysis was need. Subgroup analysis was according to the type of OP and control medication because these two factors have a great influence on the therapeutic effect of OP. If subgroup analysis failed to identify the source of heterogeneity and the number of included studies regarding an outcome was more than 10 studies, we should perform meta-regression to further explore the cause of high heterogeneity. Sample size, treatment course, year of publication and age were common variables that cause heterogeneity, therefore, the meta-regression analysis for sample size, treatment course, year of publication and age was performed to determine the possible sources of inter-study heterogeneity. Otherwise, we used a fixed-effects model (*p* ≥ 0.05 or *I* ^2^ ≤ 50%). We conducted a sensitivity investigation by excluding the individual articles one by one to test the strength and stability of the pooled data. Besides, the publication bias effect was examined using the Egger’s test. We computed the relative risk (RR) and the standard mean difference (SMD) for dichotomous outcomes and continuous outcomes, respectively.

## Results

### Description of Studies

**FIGURE 1:**
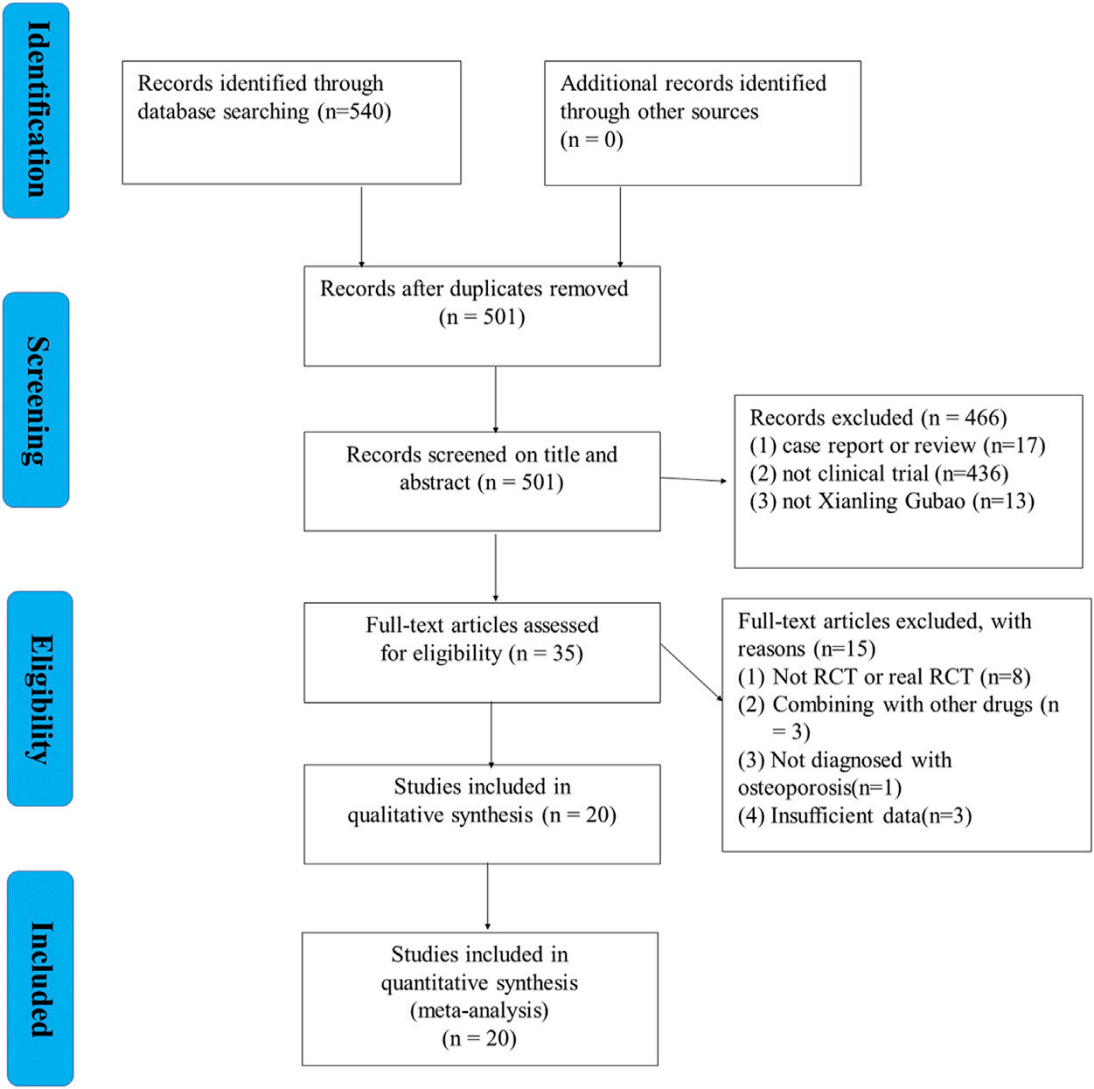
Flowchart of study selection.

Overall, 540 relevant articles were identified from the eight databases. Next, 501 articles were left after elimination of duplicates. Afterwards, 466 articles were removed after detailed screening of titles of the abstracts. Thorough screening of the full text of the 35 studies was done, of which 15 were removed for not meeting the study inclusion criteria. Eight studies (Wei, 2013; Tan, et al., 2015; Zhou et al., 2016; Wang and Wang, 2017; Li, 2018; Li et al., 2018; Dang, 2019; Ni and Xiao, 2019) were removed for not RCT or real RCT. Three studies (Li, 2020; Lv, 2020; Xu et al., 2020) were removed for combining with other drugs. One study (Jiang, 2006)was removed for not diagnosed with OP. Three studies (Fang et al., 2001; Gong et al., 2012; Yu, 2014) were removed for insufficient data. Finally, 20 articles (Xu, et al., 2009; Zhuang, 2013; Fang, 2014; Liu, 2014; Bao and Lin, 2015; Bao and Li, 2015; Xu et al., 2015; Han, 2016; Hou et al., 2016; Liu and Bai, 2016; Zhang, 2017; Feng et al., 2018; Liu, 2018; Yan, 2018; Ye et al., 2018; Li et al., 2019; Kang et al., 2020; Wang F. L. et al., 2020; Zhang et al., 2020; Zhou, 2020) were included for analysis. Figure 1 illustrates the flow diagram of the search criteria, as well as the selection process in detail.

### General Characteristics of the Included Studies

Table 1 summarizes the characteristics of the all the enrolled RCTs. They were published between 2009 and 2020. Overall, the RCTs involved 1911 participants, 949 in the experimental group vs. 962 in the control group. All the studies examined the effect of XLGB combined with ALE on POP, eight studies (Liu, 2014; Bao and Li, 2015; Han, 2016; Liu and Bai, 2016; Liu, 2018; Li et al., 2019; Kang et al., 2020; Zhang et al., 2020) for senile OP, three studies (Xu, et al., 2009; Zhou, 2020; Zhuang, 2013) for postmenopausal OP and the others were unreported. In 11 of the included studies (Zhuang, 2013; Fang, 2014; Liu, 2014; Bao and Li, 2015; Bao and Lin, 2015; Han, 2016; Liu and Bai, 2016; Feng et al., 2018; Yan, 2018; Ye et al., 2018; Li et al., 2019; Zhou, 2020), calcium carbonate D3 was combined to both groups. Salmon calcitonin was combined to both groups in two studies (Zhang et al., 2007; Kang et al., 2020). The enrolled studies involved at least 3 months of the intervention except one study (Liu, 2018). The composition of XLGB of our included was shown in Table 2. The composition of XLGB was same in all included studies in our study. The composition of XLGB included *Herba Epimedii* (*Epimedium brevicornu* Maxim, Yinyanghuo), *Radix Dipsaci* (*root of Dipsacus asper* Wall et Henry, Xuduan), *Fructus Psoraleae* (*Fruit of Psoralea corylifolia* Linn, Buguzhi), *Rhizoma Anemarrhenae* (*rhizome of Anemarrhena asphodeloides* Bunge, Zhimu), *Radix et Rhizoma Salviae* (*root and rhizome of Salvia miltiorrhiza* Bunge, Danshen), and *Radix Rehmanniae* (*root of Rehmannia glutinosa (Gaertn.)* DC, Dihuang).

**TABLE 1:** The characteristics of the included studies.

Study	Study design	Sample size	Osteoporosis type	Sample and mean age		Interventions		Course of treatment	Outcome index
				EG	CG	EG	CG		
Zhou (2020)	RCT	126	Postmenopausal osteoporosis	63; 66.79 years	63; 66.72 years	XLGB (1.0 g, tid) + CG	1. Alendronate (70 mg, qw)	6 months	ALP, S-Ca, BGP, BMD-LS, BMD-FN, BMD-WA, VAS
							2. Calcium carbonate D3 (600 mg, bid)		
Zhang et al. (2020)	RCT	86	Senile osteoporosis	43; 71.93 years	43; 71.54 years	XLGB (1.5 g, tid) + CG	Alendronate (70 mg, qw)	12 months	ALP, BMD-LS, BMD-FN, VAS, ER
Wang et al. (2020a)	RCT	92	Primary osteoporosis	46; 67.95 years	46; 68.75 years	XLGB (1.0 g, tid) + CG	Alendronate (10 mg, qd)	3 months	ALP, BGP, BMD-LS, BMD-FN, ER
Kang et al. (2020)	RCT	64	Senile osteoporosis	32; 66.87 years	32; 66.91 years	XLGB (1.5 g, bid) + CG	1. Alendronate (10 mg, qd)	3 months	ALP, BGP, BMD-LS, BMD-FN, ADR, ER
							2. Salmon calcitonin (8.3 μg, qd)		
Li et al. (2019)	RCT	124	Senile osteoporosis	62; 75 years	62; 76 years	XLGB (1.5 g, bid) + CG	1. Alendronate (70 mg, qw)	6 months	ALP, S-P, BMD-LS, ER
							2. Calcium carbonate D3 (600 mg, qd)		
Feng et al. (2018)	RCT	75	Primary osteoporosis	32; NA	43; NA	XLGB (1.0 g, tid) + CG	1. Alendronate (70 mg, qw)	12 months	ALP, S-Ca, S-P, BMD-LS, BMD-FN
							2. Calcium carbonate D3 (600 mg, bid)		
Liu (2018)	RCT	66	Senile osteoporosis	33; 68.6 years	33; 67.3 years	XLGB (1.5 g, bid)+CG	Alendronate (10 mg, qd)	1 month	BGP, BMD-LS, ADR
Ye et al. (2018)	RCT	134	Primary osteoporosis	67; 58.93 years	67; 59.37 years	XLGB (1.0 g, tid) + CG	1. Alendronate (70 mg, qw)	6 months	ALP, BGP, BMD-LS, BMD-FN, ER
							2. Calcium carbonate D3 (600 mg, qd)		
Yan (2018)	RCT	68	Primary osteoporosis	34; 63.2 years	34; 63.5 years	XLGB (1.0 g, tid) + CG	1. Alendronate (70 mg, qw)	6 months	BGP, BMD-LS, BMD-FN
							2. Calcium carbonate D3 (600 mg, bid)		
Zhang (2017)	RCT	100	Primary osteoporosis	50; 70.23 years	50; 70.15 years	XLGB (1.5 g, bid) + CG	1. Alendronate (70 mg, qw)	12 months	ALP, S-Ca, S-P, BGP, BMD-LS, ADR, ER
							2. Salmon calcitonin (50U, qw)		
Hou et al. (2016)	RCT	112	Primary osteoporosis	56; 67.73 years	56; 69.13 years	XLGB (1.5 g, bid) + CG	Alendronate (10 mg, qd)	3 months	ALP, S-Ca, S-P, BGP, BMD-LS, BMD-FN, ER
Han (2016)	RCT	110	Senile osteoporosis	55; 72.1 y	55; 71.9 years	XLGB (1.5 g, bid) + CG	1. Alendronate (70 mg, qw)	6 months	ALP, S-Ca, S-P, ER
							2. Calcium carbonate D3 (600 mg, qd)		
Liu and Bai (2016)	RCT	150	Senile osteoporosis	74; NA	76; NA	XLGB (1.0 g, bid) + CG	1. Alendronate (70 mg, qw)	6 months	BMD-LS, VAS, ADR, ER
							2. Calcium carbonate D3 (600 mg, qd)		
Xu et al. (2015)	RCT	90	Primary osteoporosis	45; NA	45; NA	XLGB (1.0 g, bid)+CG	Alendronate (70 mg, qw)	6 months	BMD-LS, VAS, ER
Bao and Li (2015)	RCT	64	Senile osteoporosis	32; NA	32; NA	XLGB (1.5 g, bid) + CG	1. Alendronate (70 mg, qw)	12 months	ALP, S-Ca, S-P, BMD-LS, ER
							2. Calcium carbonate D3 (600 mg, qd)		
Bao and Lin (2015)	RCT	140	Primary osteoporosis	70; 59.0 years	70; 58.5 years	XLGB (1.5 g, bid) + CG	1. Alendronate (70 mg, qw)	6 months	BMD-LS, ER
							2. Calcium carbonate D3 (500 mg, qd)		
Fang (2014)	RCT	80	Primary osteoporosis	40; 62.68 years	40; 63.58 years	XLGB (1.0 g, tid) + CG	1. Alendronate (70 mg, qw)	12 months	ALP, BGP, BMD-LS, ER
							2. Calcium carbonate D3 (600 mg, qd)		
Liu (2014)	RCT	62	Senile osteoporosis	31; NA	31; NA	XLGB (1.5 g, bid) + CG	Alendronate (10 mg, qd)	6 months	BMD-FN, ADR, ER
Zhuang (2013)	RCT	64	Postmenopausal osteoporosis	32; 59.78 years	32; 60.34 years	XLGB (1.5 g, bid) + CG	1. Alendronate (70 mg, qw)	6 months	BMD-LS, BMD-FN, VAS, ADR
							2. Calcium carbonate D3 (600 mg, qd)		
Xu et al. (2009)	RCT	104	Postmenopausal osteoporosis	52; NA	52; NA	XLGB (1.5 g, tid) + CG	Alendronate (70 mg, qw)	6 months	BMD-LS, BMD-WA, VAS, ADR

RCT, randomized controlled trial; EG, experimental group; CG, control group; XLGB, Xianling GuBao Capsule; ALP, alkaline phosphatase; BGP, bone gla protein; S-Ca, serum calcium; S-P, serum phosphorus; BMD-LS, bone mineral density-lumbar spine; BMD-FN, bone mineral density-femoral neck; BMD-WA, bone mineral density-Ward’s area; VAS, Visual Analog Score; ADR, adverse drug reaction; ER, effective rate; NA, not available; qd, once a day; bid, twice a day; tid, three times a day; qw, once a week; y, year.

**TABLE 2:** The composition of XLGB capsule.

Study	Formulation	Source	Species	Quality control reported? (Y/N)	Chemical analysis reported? (Y/N)
All the included studies	XLGB capsule (0.5 g)	Sinopharm group tongjitang pharmaceutical Co., Ltd.	*Epimedium sagittatum* (Siebold and Zucc.) maxim [*Berberidaceae*; Herba Epimedii]; *Dipsacus asper* wall. Ex DC [*Caprifoliaceae*; *Radix Dipsaci*]; *Cullen corylifolium* (L.) medik [*Fabaceae*; Fructus Psoraleae]; *Anemarrhena asphodeloides* Bunge [*Asparagaceae*; Rhizoma Anemarrhenae]; *Salvia miltiorrhiza* Bunge [*Lamiaceae*; Salviae miltiorrhizae radix et rhizoma]; *Rehmannia glutinosa* (Gaertn.) DC. [*Orobanchaceae*; Radix Rehmanniae]	Y- prepared according to Chinese pharmacopeia	Y-HPLC

### Risk of Bias

**FIGURE 2:**
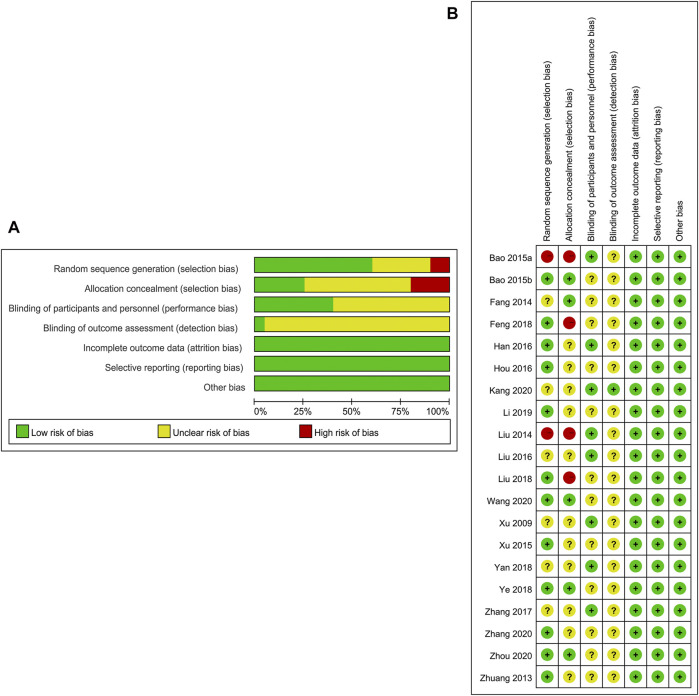
**(A)** Risk of bias graph: review authors’ judgements about each risk of bias item presented as percentages across all included studies. **(B)** Risk of bias summary: review authors' judgements about each risk of bias item for each included study.

The Cochrane risk of bias tool was employed to explore the risk of bias. Of the 20 enrolled articles, the criteria number varied from 7/7 to 4/7. Besides, 12 of the enrolled RCTs (Bao and Li, 2015; Feng et al., 2018; Han, 2016; Hou et al., 2016; Li et al., 2019; Liu, 2018; Wang, et al., 2020a; Xu et al., 2015; Ye et al., 2018; Zhang et al., 2020; Zhou, 2020; Zhuang, 2013) had documented the precise approach employed in random sequences generation. Five studies (Fang, 2014; Bao and Li, 2015; Ye et al., 2018; Wang Y. et al., 2020; Zhou, 2020) mentioned the concealment allocation. Eight studies (Bao and Li, 2015; Han, 2016; Kang et al., 2020; Liu, 2014; Liu and Bai, 2016; Xu, et al., 2009; Yan, 2018; Zhang, 2017) reported the blinding, the residual studies remained unclear. All the RCTs met the incomplete outcome data criterion as no drop-out patients or drop-out data were reported specifically. No studies had risk of bias in selective reporting. Baseline comparisons were present and the consent of the participants were well documented, and other biases were not present in all the enrolled studies. More details about risk of bias evaluation of each trial are indicated in Figure 2.

### Results of Meta-analysis

#### ALP

**FIGURE 3:**
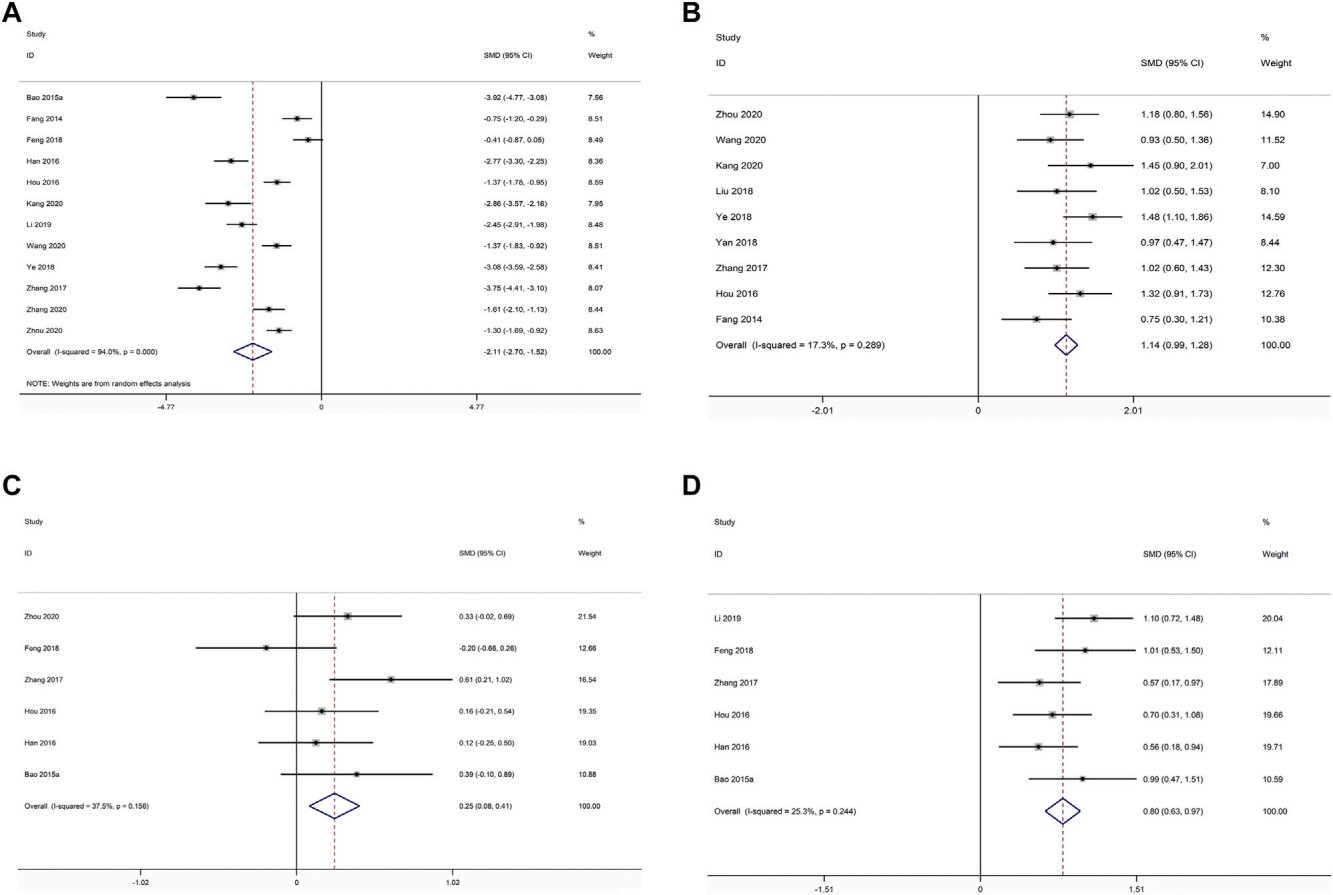
Forest plot of XLGB combined with ALE vs. ALE alone with regard to ALP **(A)**, BGP **(B)**, S-Ca **(C)**, and S-P **(D)**.

**FIGURE 4:**
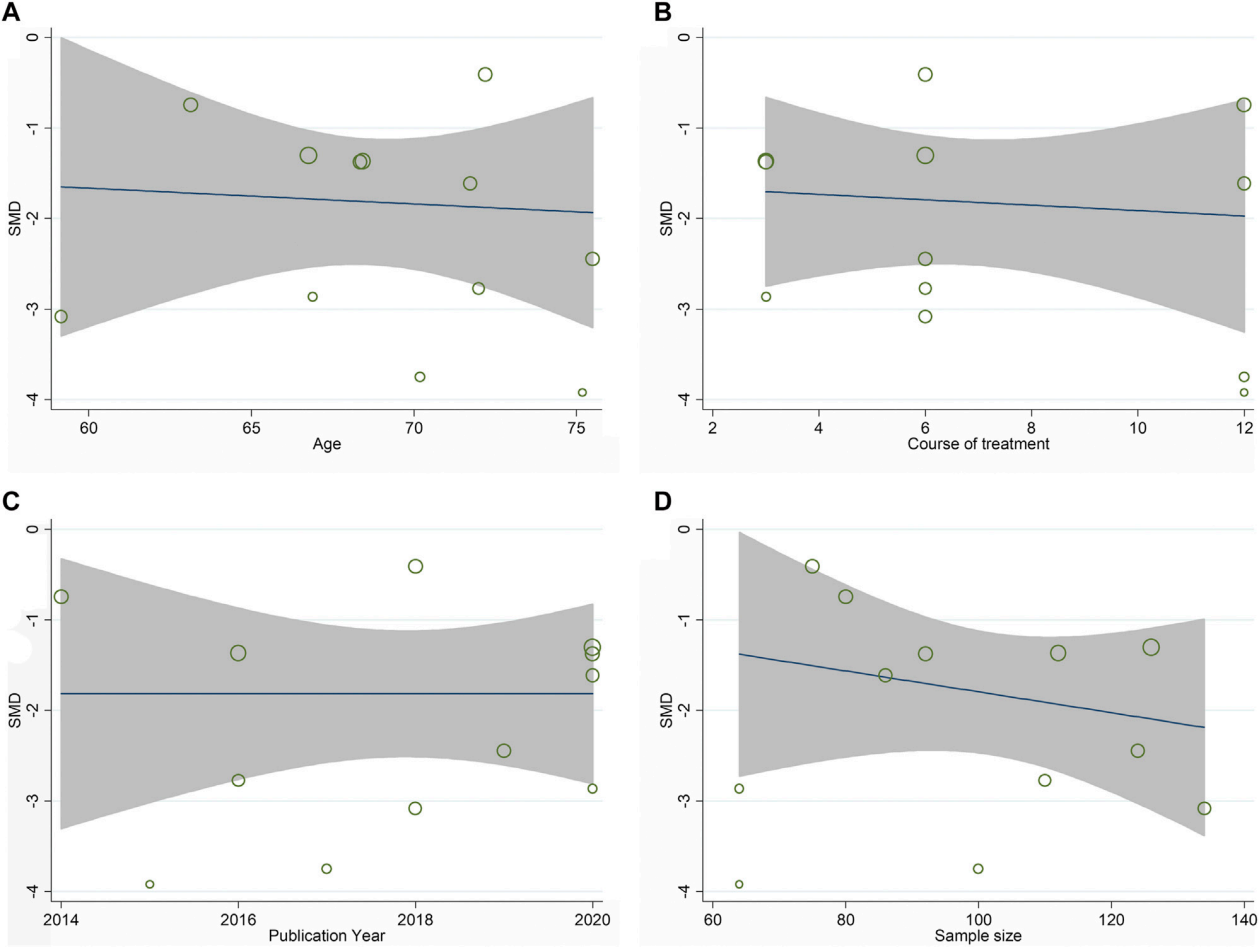
Meta-regression analysis of ALP: **(A)** Age **(B)** Course of treatment **(C)** Publication year **(D)** Sample size.

12 studies compared XLGB plus ALE with ALE regarding ALP (Fang, 2014; Bao and Li, 2015; Han, 2016; Hou et al., 2016; Zhang, 2017; Feng et al., 2018; Ye et al., 2018; Li et al., 2019; Wang Y. et al., 2020; Kang et al., 2020; Zhang et al., 2020; Zhou, 2020). As illustrated in Figure 3A, the pooled results deminstrated that XLGB plus ALE was remarkable for reducing ALP in contrast with ALE alone (SMD = −2.107; 95% CI = −2.695 to −1.519; *p* < 0.001; heterogeneity χ^2^ = 182.65, df = 11, *I* ^2^ = 94.0%, *p* < 0.001). Meta-regression was employed to explore heterogeneity sources. The meta-regression analysis for sample size, treatment course, year of publication and age was performed to determine the possible sources of inter-study heterogeneity (Figure 4). Overall, the sample size (*β* = −0.002; *p* = 0.881; Adj *R* ^2^ = −10.30%), course of treatment (*β* = −0.071; *p* = 0.479; Adj *R* ^2^ = −5.26%), publication year (*β* = 0.065; *p* = 0.709; Adj *R* ^2^ = −9.46%) and age (*β* = −0.037; *p* = 0.632; Adj *R* ^2^ = −8.37%) were not the remarkable sources of heterogeneity for ALP.

#### BGP

Nine studies comparing XLGB plus ALE with ALE for BGP (Fang, 2014; Hou et al., 2016; Zhang, 2017; Liu, 2018; Yan, 2018; Ye et al., 2018; Wang Y. et al., 2020; Kang et al., 2020; Zhou, 2020). The available data illustrated that XLGB plus ALE remarkably increased the BGP in contrast with ALE alone (SMD = 1.136; 95% CI = 0.990 to 1.283; *p* < 0.001; heterogeneity χ^2^ = 9.67, df = 8, *I* ^2^ = 17.3%, *p* = 0.289, Figure 3B).

#### S-Ca

Six studies reported XLGB plus ALE vs. ALE according to S-Ca (Bao and Li, 2015; Han, 2016; Hou et al., 2016; Zhang, 2017; Feng et al., 2018; Zhou, 2020). The pooled results illustrated that XLGB and ALE combination was remarkable for raising S-Ca in contrast with ALE alone (SMD = 0.247; 95% CI = 0.083 to 0.410; *p* = 0.003, heterogeneity χ^2^ = 8.00, df = 5, *I* ^2^ = 37.5%, *p* = 0.156, Figure 3C).

#### S-P

There were six studies comparing XLGB plus ALE with ALE about the S-P (Bao and Li, 2015; Han, 2016; Hou et al., 2016; Zhang, 2017; Feng et al., 2018; Li et al., 2019). The pooled results exhibited that XLGB and ALE combination remarkably increased S-P in contrast with ALE alone (SMD = 0.797; 95% CI = 0.627 to 0.966; *p* < 0.001, heterogeneity χ^2^ = 6.70, df = 5, *I* ^2^ = 25.3%, *p* = 0.244, Figure 3D).

#### VAS

**FIGURE 5:**
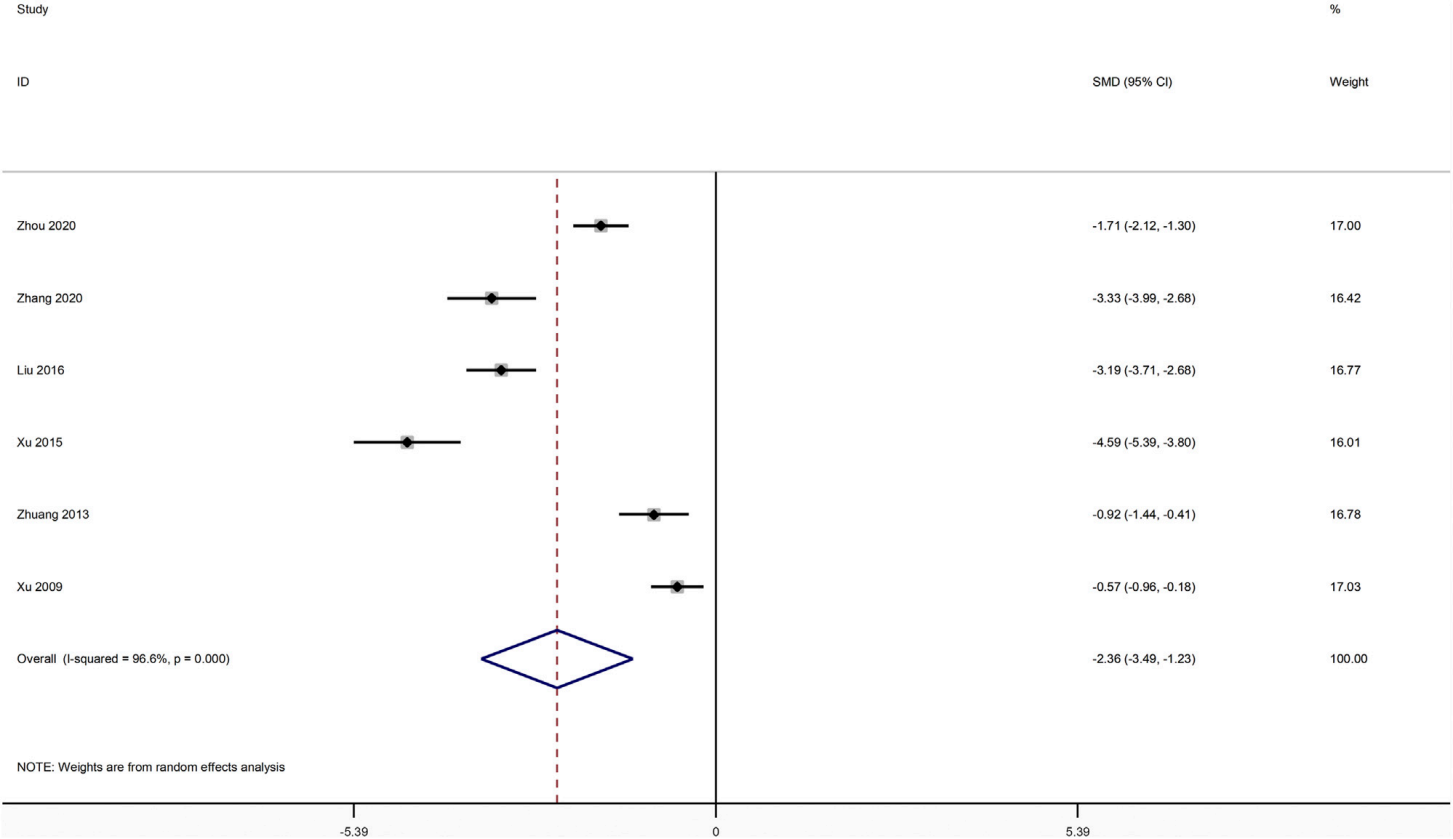
Forest plot of XLGB combined with ALE vs. ALE alone with regard to VAS.

Six studies compared XLGB plus ALE with ALE with regards to VAS(Liu and Bai, 2016; Xu et al., 2015; Xu, et al., 2009; Zhang et al., 2020; Zhou, 2020; Zhuang, 2013). As illustrated in Figure 5, the pooled results exhibited that XLGB plus ALE was remarkable for reducing VAS in contrast with ALE alone (SMD = −2.361; 95% CI = −3.490 to −1.232; *p* < 0.001; heterogeneity χ^2^ = 144.99, df = 5, *I* ^2^ = 96.6%, *p* < 0.001).

#### BMD

##### BMD at Lumbar Spine

**FIGURE 6:**
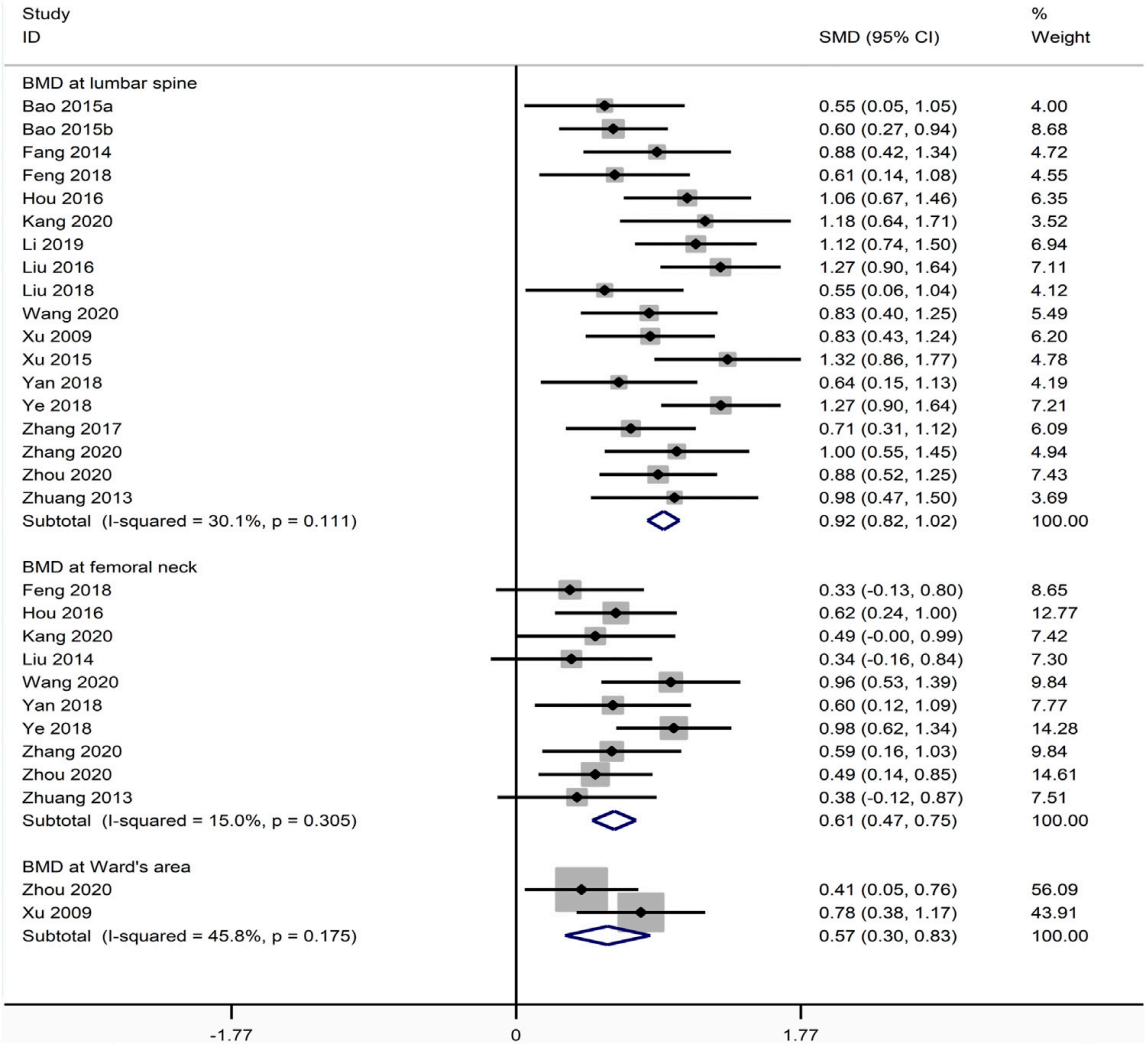
Forest plot of XLGB combined with ALE vs. ALE alone with regard to BMD at lumbar spine **(A)** BMD at femoral neck **(B)** and BMD at Ward’s area **(C)**.

18 compared XLGB plus ALE with ALE about the BMD at lumbar spine (Xu, et al., 2009; Zhuang, 2013; Fang, 2014; Bao and Lin, 2015; Bao and Li, 2015; Xu et al., 2015; Hou et al., 2016; Liu and Bai, 2016; Zhang, 2017; Feng et al., 2018; Liu, 2018; Yan, 2018; Ye et al., 2018; Li et al., 2019; Kang et al., 2020; Wang F. L. et al., 2020; Zhang et al., 2020; Zhou, 2020). As illustrated in Figure 6A, the pooled results depicted that XLGB plus ALE was remarkable for improving BMD at lumbar spine in contrast with ALE alone (SMD = 0.917; 95% CI = 0.817 to 1.016; *p* < 0.001; heterogeneity χ^2^ = 24.31, df = 17, *I* ^2^ = 30.1%, *p* = 0.111).

##### BMD at Femoral Neck

10 studies reported XLGB plus ALE with ALE with regards to BMD at femoral neck (Zhuang, 2013; Liu, 2014; Hou et al., 2016; Feng, Huang and Qu, 2018; Yan, 2018; Ye et al., 2018; Wang F. L. et al., 2020; Kang et al., 2020; Zhang et al., 2020; Zhou, 2020). The pooled data illustrated that XLGB plus ALE was remarkable for lifting BMD at femoral neck in contrast with ALE alone (SMD = 0.610; 95% CI = 0.475 to 0.746; *p* < 0.001, heterogeneity χ^2^ = 10.58, df = 9, *I* ^2^ = 15.0%, *p* = 0.305, Figure 6B).

##### BMD at Ward’s Area

Only two studies compared XLGB plus ALE with ALE as for BMD at Ward’s area (Xu, et al., 2009; Zhou, 2020). The available data demonstrated that XLGB plus ALE remarkably raised BMD at Ward’s area relative to ALE alone (SMD = 0.569; 95% CI = 0.304 to 0.833; *p* < 0.001; heterogeneity χ^2^ = 1.84, df = 1, *I* ^2^ = 45.8%, *p* = 0.175, Figure 6C).

#### Effective Rate

**FIGURE 7:**
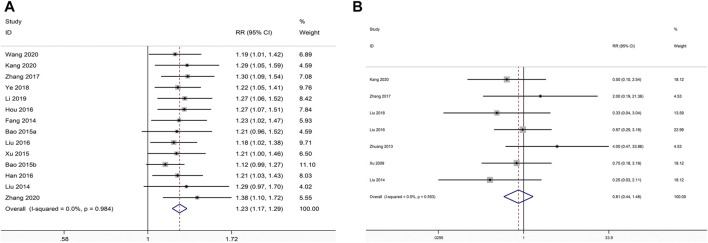
Forest plot of XLGB combined with ALE vs. ALE alone with regard to effective rate **(A)** and ADR **(B)**.

There were 14 studies comparing XLGB plus ALE with ALE about the effective rate (Fang, 2014; Liu, 2014; Bao and Li, 2015; Bao and Lin, 2015; Xu et al., 2015; Han, 2016; Hou et al., 2016; Liu and Bai, 2016; Zhang, 2017; Ye et al., 2018; Li et al., 2019; Wang Y. et al., 2020; Kang et al., 2020; Zhang et al., 2020). The pooled data illustrated that the combination of XLGB with ALE remarkably improved effective rate in relative to ALE alone (RR = 1.230; 95% CI = 1.173 to 1.290; *p* < 0.001, heterogeneity χ^2^ = 4.55, df = 13, I^2^ = 0%, *p* = 0.984, Figure 7A).

#### ADR

We identified ADR in seven studies (Kang et al., 2020; Liu, 2014; Liu and Bai, 2016; Liu, 2018; Xu, et al., 2009; Zhang, 2017; Zhuang, 2013). The frequency of adverse events was 18/297 in the trial group and 22/295 in the control group. In the pooled data, the rate of ADR was not remarkably different between the two groups (RR = 0.812; 95% CI = 0.444 to 1.485; *p* = 0.499, heterogeneity χ^2^ = 4.93, df = 6, *I* ^2^ = 0%, *p* = 0.553, Figure 7B). Our results revealed that gastrointestinal discomfort, liver function damage and menstruation disorders or amenorrhea frequently constitute the most frequently occurring adverse events. Remarkable adverse impacts were mild, with no severe adverse impacts, including life threatening reported in the included RCTs.

### Subgroup Analysis

As results of ALP and VAS showed high heterogeneity in our study, we performed subgroup analysis according to the type of OP and control medication in Table 3. However, the results of subgroup analysis revealed that the type of OP and control medication were not the remarkable sources of heterogeneity for ALP and VAS.

**TABLE 3:** Subgroup analysis.

Outcome	Subgroup factor	Number of study	Cases (EG/CG)	*I* ^2^ (%)	Heterogeneity (P)	Pooling model	Z test (P)
ALP	—						
	OP type	—	—	—	—	—	—
	Senile OP	6	274/274	86.9	<0.0001	Random	<0.0001
	Primary OP	5	241/252	94.2	<0.0001	Random	0.001
	Postmenopausal OP	1	63/63	—	—	Fixed	<0.0001
	Control medication	—	—	—	—	—	—
	ALE alone	3	145/145	0	0.710	Fixed	<0.0001
	ALE combined with OP basic treatment	9	433/444	95.3	<0.0001	Random	<0.0001
VAS	—	—	—	—	—	—	—
	OP type	—	—	—	—	—	—
	Senile OP	2	110/108	0	0.742	Fixed	<0.0001
	Primary OP	1	45/45	—	—	Fixed	<0.0001
	Postmenopausal OP	3	147/147	87.4	<0.0001	Random	0.003
	Control medication	—	—	—	—	—	—
	ALE alone	3	140/140	98.1	<0.0001	Random	0.029
	ALE combined with OP basic treatment	3	162/160	94.8	<0.0001	Random	0.002

OP, osteoporosis; ALE, alendronate; EG, experimental group; CG, control group; ALP, alkaline phosphatase; VAS, Visual Analog Score.

### Publication Bias and Sensitivity Analysis

**FIGURE 8:**
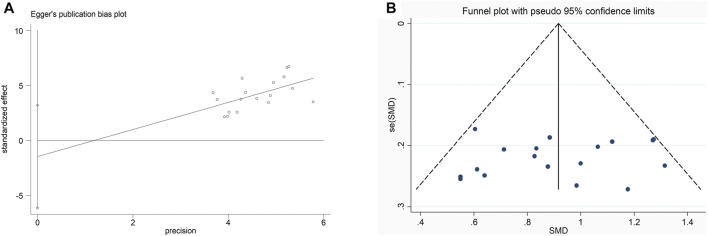
Funnel plot and Egger’s test of BMD at lumbar spine.

We used the funnel plots along with Egger’s test (Figure 8) to examine the possible publication bias of the BMD at lumbar spine in this meta-analysis. Consequently, the symmetrical shape of the funnel plots, as well as the *p* values from Egger’s tests, revealed that there was no remarkable publication bias for BMD at lumbar spine (*p* = 0.515).

**FIGURE 9:**
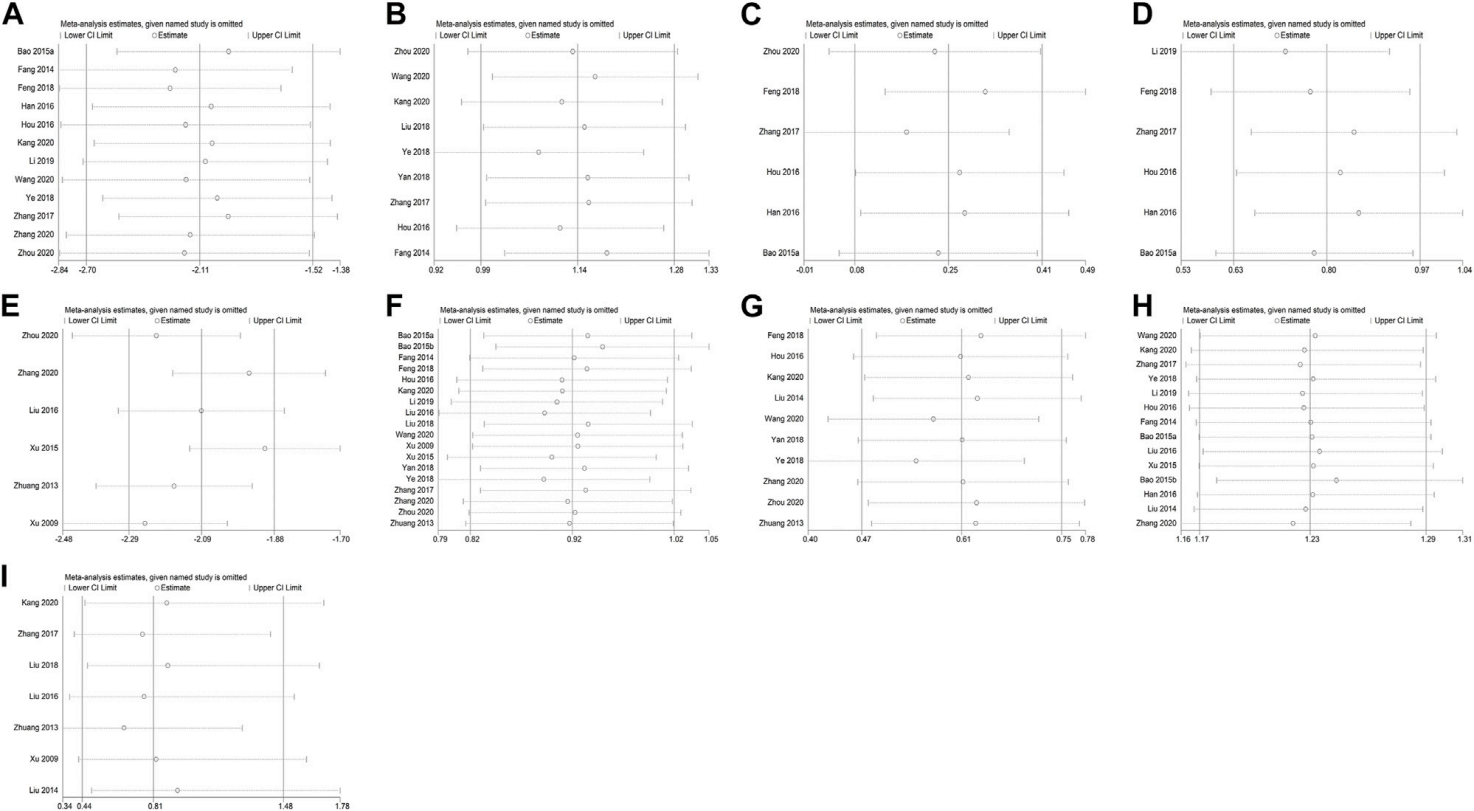
Sensitivity analysis for ALP **(A)**, BGP **(B)**, S-Ca **(C)**, S-P **(D)**, VAS **(E)**, BMD at lumbar spine **(F)**, BMD at femoral neck **(G)**, effective rate **(H)**, and ADR **(I)**.

To establish the impact of each included study on the pooled data of ALP, BGP, S-Ca, S-P, VAS, BMD at lumbar spine, BMD at femoral neck, effective rate and ADR to validate the robustness of our findings, we performed a sensitivity assessment by excluding one article at a time and computing the pooled data for the rest of the RCTs. The sensitivity investigation results revealed that there was no marked effect on pooled data after eliminating each study singly, implying that the findings of this meta-analysis are comparatively robust (Figure 9).

### GRADE Assessment

The GRADE approach was employed to explore the quality of evidence of the outcomes, which exhibited very low quality, moderate, or low with heterogeneity problem and methodological problems. Table 4 illustrates the GRADE evidence profiles.

**TABLE 4:** GRADE evidence profile.

Quality assessment							No. of patients		Effect		Quality	Importance
No of studies	Design	Risk of bias	Inconsistency	Indirectness	Imprecision	Other considerations	XianLing GuBao combined with alendronate	Alendronate	Relative (95% CI)	Absolute		
Alkaline phosphatase(ALP) (Better indicated by lower values)												
12	Randomised trials	serious^1^	serious^2^	No serious indirectness	No serious imprecision	None	578	589	—	SMD 2.09 lower (2.67–1.51 lower)	⊕⊕○○LOW	CRITICAL
Serum-calcium(S-Ca) (better indicated by lower values)												
6	Randomised trials	Very serious^1^ ^,^ ^3^	No serious inconsistency	No serious indirectness	No serious imprecision	None	288	299	—	SMD 0.24 higher (0.08–0.41 higher)	⊕⊕○○LOW	IMPORTANT
Bone gla protein(BGP) (better indicated by lower values)												
9	Randomised trials	Very serious^1^ ^,^ ^4^	No serious inconsistency	No serious indirectness	No serious imprecision	None	421	421	—	SMD 1.13 higher (0.98–1.27 higher)	⊕⊕○○LOW	CRITICAL
Serum-phosphorus(S-P) (better indicated by lower values)												
6	Randomised trials	Very serious^1^ ^,^ ^4^	No serious inconsistency	No serious indirectness	No serious imprecision	None	287	298	—	SMD 0.79 higher (0.62–0.96 higher)	⊕⊕○○LOW	IMPORTANT
Visual analogue scale(VAS) (better indicated by lower values)												
6	Randomised trials	Very serious^1^ ^,^ ^3^	serious^2^	No serious indirectness	No serious imprecision	None	302	300	—	SMD 2.34 lower (3.46–1.22 lower)	⊕○○○VERY LOW	IMPORTANT
BMD-LS(Bone mineral density-lumbar spine) (better indicated by lower values)												
18	Randomised trials	serious^1^	No serious inconsistency	No serious indirectness	No serious imprecision	None	856	865	—	SMD 0.91 higher (0.81–1.01 higher)	⊕⊕⊕○MODERATE	CRITICAL
BMD-FN(Bone mineral density-femoral neck) (better indicated by lower values)												
10	Randomised trials	serious^1^	No serious inconsistency	No serious indirectness	No serious imprecision	None	436	447	—	SMD 0.61 higher (0.47–0.74 higher)	⊕⊕⊕○MODERATE	CRITICAL
BMD-WA(Bone mineral density-Ward's area) (better indicated by lower values)												
2	Randomised trials	serious^4^	serious^2^	No serious indirectness	No serious imprecision	None	115	115	—	SMD 0.56 higher (0.3–0.83 higher)	⊕⊕○○LOW	CRITICAL
Adverse drug reaction(ADR)												
7	Randomised trials	Very serious^1^ ^,^ ^4^	No serious inconsistency	No serious indirectness	No serious imprecision	None	18/297 (6.1%)	22/295 (7.5%)	RR 0.81 (0.44–1.48)	14 fewer per 1,000 (from 42 fewer to 36 more)	⊕⊕○○LOW	CRITICAL
								7.7%		15 fewer per 1,000 (from 43 fewer to 37 more)		
Effective rate(ER)												
14	Randomised trials	serious^4^	No serious inconsistency	No serious indirectness	No serious imprecision	None	644/696 (92.5%)	522/694 75.2%)	RR 1.23 (1.17–1.29)	173 more per 1,000 (from 128 more to 218 more)	⊕⊕⊕○MODERATE	CRITICAL
								75.3%		173 more per 1,000 (from 128 more to 218 more)		

CI, Confidence interval; RR, Risk ratio; GRADE Working Group grades of evidence: High quality: Further research is very unlikely to change our confidence in the estimate of effect. Moderate quality: Further research is likely to have an important impact on our confidence in the estimate of effect and may change the estimate. Low quality: Further research is very likely to have an important impact on our confidence in the estimate of effect and is likely to change the estimate. Very low quality: We are very uncertain about the estimate.

1 Many of the included studies lack of allocation concealment.

2 Heterogeneity (*I* ^2^ > 50%, *p* < 0.05) was found.

3 No details of random protocol were reported.

4 Many of the included studies lacked of reporting the implementation of blinding.

## Discussion

OP is a chronic bone disease that manifests through low bone mass, as well as structural degeneration of bone tissue. Presently, the medications applied in the clinical treatment of OP, such as estrogen, calcitonin, selective estrogen modulator, and bisphosphonates are primarily conducive for mitigating bone absorption (Liu, et al., 2020). As clinical practice and research develop, accumulating evidence shows that Chinese herbal medicine have a remarkable effect on the prevention and treatment of OP (Chen, et al., 2017a; Lin, et al., 2017; Luo et al., 2017; Shi, et al., 2017). XLGB is a commonly used Chinese herbal formula, has been widely used in OP, osteoarthritis, aseptic necrosis of head of femur and osteoporotic fracture in China (Ni et al., 2011). Modern pharmacological studies have shown that XLGB can regulate body metabolism, inhibit osteoclast activity, reduce bone calcium loss, thereby increase bone density and effectively relieve pain (Qin et al., 2015). Besides, a previous study showed that XLGB also has the ability to anti-inflammatory, repair bone damage and restore bone structure (Ma, 2018). ALE, a second generation bisphosphonate, represses osteoclast activity, diminishes the resorption of bone tissue and maintains the balance of bone formation and resorption (Lin and Lane, 2003; Madore et al., 2004). Nonetheless, no systematic review and meta-analysis on the safety and efficacy of XLGB combined with ALE in the treatment of POP has been demonstrated. Therefore, herein, we performed this systematic review and meta-analysis to explore the safety and effect of XLGB combined ALE in individuals with POP to provide evidence for clinical practice, as well as scientific research.

### Summary of Evidence

This study is the first systematic review and meta-analysis of the efficacy and safety of XLGB combined with ALE for POP. Twenty high-quality RCTs involving 1911 individuals with POP were enrolled in the analysis. The primary findings of present systematic review illustrated that XLGB combined with ALE could remarkably increase the BGP, S-Ca, S-P, BMD and highly improve the total effective rate in POP patients. Meanwhile, the ALP and VAS were obviously reduced, suggesting that XLGB can effectively relieve the pain which caused by OP. However, no remarkable difference was found in ADR between the two groups, which indicated that XLGB was relatively safe and well tolerated for individuals with POP. Therefore, we provide supporting evidence that, to a remarkable extent, XLGB can potentially be recommended for scheduled use for POP patients. However, ALP and VAS represented high heterogeneity in our meta-analysis, but we failed to find out the source of heterogeneity through subgroup analysis and meta-regression. The data of gender ratio was missing in most of our included studies, thus, we could not pool the data. Gender is an important factor in the incidence of OP. Therefore, we speculated that gender may contribute to the cause of unresolved heterogeneity. Besides, not all our included studies reported the generation of random allocation and the method randomization varied between the included studies. The blinding procedure was not reported or remained unclear in some studies and the method of blinding also varied. This also may be the source of unresolved heterogeneity, which would be figured out by more RCTs with high quality in the future.

### Comparison With Previous Studies

Several systematic reviews and meta-analyses have demonstrated the efficacy and safety of Chinese herbal medicine (CHM) in treating OP. A meta-analysis constituting 19 RCTs involving 1831 patients revealed that Chinese herbal compound is safe and effective in increasing BMD, improving effective rate and relieving pain in the treatment of senile OP (Fu et al., 2020). However, the S-Ca, S-P, and ALP were not remarkably different after Chinese herbal compound treatment, which was inconsistent with our results. The contradictory conclusions originated from the differences of the search strategies, data abstraction along with analyses, and the selection criteria. Numerous varieties of Chinese herbal compound and control group lead to high heterogeneity, which may be the primary cause of contradiction. The findings of another systematic review and meta-analysis indicated that Liuwei Dihuang pill is effective in improving BMD of hip and lumbar spine and alleviating pain in patients with postmenopausal OP (Wang, et al., 2020a). Besides, the findings of a previous review showed that Zuogui pill combined with anti-OP drugs remarkably increased BMD at different sites and improved the bone metabolism markers for treating OP (Li, et al., 2020). Besides, most of our findings were congruent with the data of An et al. (An, et al., 2019). They performed a meta-analysis involving 16 trials that focused on 1,492 patients, and exhibited that XLGB combined with conventional treatment obviously increased BMD, BGP as well as effective rate, reduced ALP and VAS in postmenopausal OP. Whereas, the S-P and S-Ca was not increased in their study. Therefore, our data are inconsistent with the findings of previous researches in some aspects, which could be linked to: 1) The enrolled articles in the previous meta-analyses varied considerably in quality. Nonetheless, herein, RCTs having a risk of bias score ≥4 on the basis of the Cochrane RoB tool were enrolled, implying that only high-quality RCTs were enrolled in our study. 2) The diversity of CHM and conventional treatment in the previous researches is also a major cause of the difference. Nevertheless, herein, we focused particularly on the comparison of XLGB combined with ALE (single experimental group) and ALE alone (single control group) in the treatment of OP, which can reduce the risk of heterogeneity. 3) OP is a chronic condition with diverse kinds and stages. The OP distinct kinds and stages can affect the progress of the disease along with the treatment response. 4). OP prevalence is high in women, with 1.5-fold more likely to be affected relative to men. Hence, the difference of the gender ratio between the previous researches and our study also impacts the findings. 5) The course of treatment can either impact the results. The treatment course ranged between 1 and 12 months in the previous researches and this study. Short treatment course may result in inadequate efficacy while long treatment course can result in great adverse events, which is a contradiction in terms.

### Strengths

The strengths of this meta-analysis study consisted of a clearly defined research question, which reduced the bias in the choice of the RCTs, fidelity, and consistency to a precise research approach that we designed before the meta-analysis, an in-depth search of the literature, the agreement between the two researchers regarding the entry data components, and the quality control appraisal of all the data. All of the studies included in herein constituted RCTs with a remarkable number being high quality. This helps to overcome the drawbacks of the recall or selection bias regarding non-randomized studies. Additionally, the number of trials and the overall sample size was comparatively large (20 trails with 1911 patients). We performed subgroup assessments and meta-regression evaluation to identify the origin of heterogeneity. Consequently, no publication bias was reported in this meta-analysis, and sensitivity estimation revealed that the findings of this meta-analysis are comparatively robust.

### Limitations

This study has several limitations. Firstly, although RCTs were included, the included primary studies had some intrinsic and methodological shortcomings: 1) only 12 trials had sufficient information on the generation of random allocation. 2) The blinding procedure was not reported or remained unclear in some studies, making it a challenge to bias findings unintentionally or intentionally and to help allow the credibility of study conclusions. Triple blindness is required in further trials. Secondly, OP constitutes a chronic disease that requires life-long therapies. The long-term safety and efficacy are crucial investigations in establishing the clinical effectiveness of a drug in treatment.

Nonetheless, herein, the treatment period was between 1 and 12 months. We have not determine the long-term safety of XLGB for treating OP since the duration of treatment of the included studies was short, and no dropouts were revealed in a considerable number of the included studies. Thirdly, the formula composition, dosage, administration approaches, and period of XLGB treatments differed remarkably in the primary RCTs. This clinical heterogeneousness could compromise the viability of our findings. Fourthly, we only searched for studies published in English or Chinese repositories; therefore, the potentially relevant RCTs published in other languages could have been left out. Moreover, all RCTs included in the study were conducted in China, a potential limitation to the generalizability of our findings. Fifthly, the overall evidence quality herein was low given the high risk of bias, inconsistency, as well as potential reporting bias. Therefore, there is a need for further worldwide high quality multi-center RCTs of XLGB combined with ALE for treating OP to allow data generalization worldwide.

### Implications for Research

We herein reveal important ideas that are likely to promote research in this field. Firstly, it is evident that strategies that improve the methodological quality of RCTs are urgently needed. Going forward, we recommend that guidelines, including the CONSORT 2010 statement (Cheng, et al., 2017) should be employed to establish and report RCTs for XLGB. Secondly, despite the revelation that XLGB therapy in the analyzed studies was somewhat safe for patients with OP, further investigations are needed to confirm the safety of XLGB for OP. A standard reporting format for ADR has been developed (Bian, et al., 2010), and we propose that close attention should be paid to improve the reporting of ADRs of XLGB. Thirdly, to conclusively understand the long-term safety profile of XLGB in patients with OP, clinical trials and studies incorporating a longer follow-up period are recommended. Our results suggest that XLGB combined with ALE can be alternative treatment for OP patients, nonetheless, further large clinical studies should be conducted to explore the long-term safety, efficacy, and optimized dosages of the combination use of XLGB and ALE for treating OP.

Our present study demonstrated XLGB exerted prominent anti-osteoporosis effect in POP. The potent anti-osteoporosis effect of XLGB was possibly attributed to its six bioactive compounds: *Herba Epimedii, Radix Dipsaci, Fructus Psoraleae, Rhizoma Anemarrhenae, Radix et Rhizoma Salviae*, and *Radix Rehmanniae*. Quercetin (derived from *Herba Epimedii* and *Radix et Rhizoma Salviae*) was reported to increase the bone mass and biomechanical properties of OVX rats, which is closely related to its protective effect against tumor necrosis factor-α (TNF-α)-induced impairments in bone marrow mesenchymal stem cells (Yuan, et al., 2018). Moreover, luteolin (derived from *Herba Epimedii* and *Radix et Rhizoma Salviae*) was proved to improve bone formation in glucocorticoid-induced OP by decreasing the excess production of reactive oxygen species and increasing the proliferation and differentiation of osteoblasts (Jing, et al., 2019). Kaempferol (derived from *Herba Epimedii, Rhizoma Anemarrhenae* and *Radix et Rhizoma Salviae*) has been shown to prevent osteoporosis-induced bone loss *in vivo* and *in vitro*, which results from its regulatory effect on the mammalian target of rapamycin (mTOR) pathway (Zhao, et al., 2019). Besides, anhydroicaritin (derived from *Herba Epimedii*) also exerts a suppressive effect on receptor activator for nuclear factor-κB ligand (RANKL)-induced osteoclast differentiation, which results in increased bone loss in diabetic OP (Zheng, et al., 2017). Similarly, diosgenin (derived from *Rhizoma Anemarrhenae*) was shown to enhance the BMD of OVX rats by inhibiting the expression of RANKL and promoting osteoprotegerin (Zhang, et al., 2014). These active compounds may contribute to the promising anti-osteoporosis effect of XLGB. However, the specific mechanism of XLGB and its active compounds still needs clarity.

In our study, S-Ca increased about 0.247 in XLGB combined ALE group. However, a previous study has shown that there was no significant relationship between S-Ca and BMD (Liu and He, 2011), which suggests that the increased S-Ca has no promoting effect on the treatment of OP. Another study demonstrated ALP is an important predictor of BMD reduction and its increase is related to the risk of OP. The detection of ALP is conducive to prevent OP in the early stage (Chen H. S. et al., 2017). ALP was decreased about 2.107 by XLGB in the present meta-analysis, indicating XLGB can highly enhance BMD and exert a potent anti-osteoporosis effect in clinical practice. BGP is a non-collagen protein produced and secreted by osteoblasts, which is often used as a specific marker of bone turnover and bone formation (Garnero, 2008). It can reflect the activity of osteoblasts and is of great significance for the diagnosis of OP. BGP increased about 1.136 after XLGB treatment, which further verifies the protective effect of XLGB on OP. Thus, taking together the changes of bone metabolic markers by XLGB, XLGB possesses a prominent therapeutic effect on OP and is worthy of clinical promotion.

## Conclusion

In summary, the evidence available from the present study illustrated that XLGB combined with ALE have beneficial effects on patients with OP in terms of BMD, BGP, S-Ca, S-P, and effective rate than ALE alone. Moreover, ALP and VAS were remarkably decreased. In addition, XLGB combined with ALE would not increase the rate of ADR. Therefore, the results of our study demonstrated that XLGB is a potential candidate for OP treatment. However, taking the heterogeneity along with small sample size into account, larger multi-center and high quality RCTs are critical to go a step further in illustrating the benefits of XLGB in treatment of OP.

## Data Availability

The original contributions presented in the study are included in the article/supplementary material, further inquiries can be directed to the corresponding author.

## Abbreviations

OP, osteoporosis; POP, primary osteoporosis; RCT, randomized controlled trial; EG, experimental group; CG, control group; XLGB, Xianling GuBao Capsule; ALP, alkaline phosphatase; BGP, bone gla protein; S-Ca, serum calcium; S-P, serum phosphorus; BMD-LS, bone mineral density-lumbar spine; BMD-FN, bone mineral density-femoral neck; BMD-WA, bone mineral density-Ward’s area; VAS, Visual Analog Score; ADR, adverse drug reaction; ER, effective rate

## References

[B1] Adachi J. D. Saag K. G. Delmas P. D. Liberman U. A. Emkey R. D. Seeman E. (2001). Two-year Effects of Alendronate on Bone mineral Density and Vertebral Fracture in Patients Receiving Glucocorticoids: a Randomized, Double-Blind, Placebo-Controlled Extension Trial. Arthritis Rheum. 44 (1), 202–211. 10.1002/1529-0131(200101)44:1<202::aid-anr27>3.0.co;2-w 11212161

[B2] An Y. F. Zhang Y. L. Xie Y. M. Wei W. Jiang J. J. Wang K. L. (2019). The Effectiveness of Xianling Gubao Capsule in the Treatment of Postmenopausal Osteoporosis: a Systematic Review and Meta-Analysis. Chin. J. Osteoporos. 25 (1), 47–61.

[B3] Balshem H. Helfand M. Schünemann H. J. Oxman A. D. Kunz R. Brozek J. (2011). GRADE Guidelines: 3. Rating the Quality of Evidence. J. Clin. Epidemiol. 64 (4), 401–406. 10.1016/j.jclinepi.2010.07.015 21208779

[B4] Bao D. Y. Lin G. S. (2015). Clinical Observation of Xianling Gubao Capsule Combined with Alendronate Sodium Tablets in the Treatment of Osteoporosis. J. New Chin. Med. 47 (1), 133–134.

[B5] Bao J. G. Li X. C. (2015). Comparative Study on the Curative Effect of Xianling Gubao Capsule Combined with Calci D and Alendronate Sodium Tablets in the Treatment of Senile Osteoporosis. Liaoning J. Traditional Chin. Med. 42 (4), 784–785.

[B6] Bian Z.-X. Tian H.-Y. Gao L. Shang H.-C. Wu T.-X. Li Y.-P. (2010). Improving Reporting of Adverse Events and Adverse Drug Reactions Following Injections of Chinese Materia Medica. J. Evid. Based Med. 3 (1), 5–10. 10.1111/j.1756-5391.2010.01055.x 21349034

[B7] Bolland M. J. Grey A. B. Gamble G. D. Reid I. R. (2010). Effect of Osteoporosis Treatment on Mortality: a Meta-Analysis. J. Clin. Endocrinol. Metab. 95 (3), 1174–1181. 10.1210/jc.2009-0852 20080842

[B8] Burch J. Rice S. Yang H. Neilson A. Stirk L. Francis R. (2014). Systematic Review of the Use of Bone Turnover Markers for Monitoring the Response to Osteoporosis Treatment: the Secondary Prevention of Fractures, and Primary Prevention of Fractures in High-Risk Groups. Health Technol. Assess. 18 (11), 1–180. 10.3310/hta18110 PMC4781229 24534414

[B9] Chen B. Wang L. Li L. Zhu R. Liu H. Liu C. (2017a). Fructus Ligustri Lucidi in Osteoporosis: A Review of its Pharmacology, Phytochemistry, Pharmacokinetics and Safety. Molecules 22 (9), 1469. 10.3390/molecules22091469 PMC6151717 28872612

[B10] Chen H. S. Hu F. Liu D. M. Feng J. Zhao C. F. (2017b). The Relationship between Bone mineral Density and Blood Calcium, Blood Phosphorus and Bone Turnover Indexes in Middle-Aged and Elderly People. Chin. J. Gerontol. 37, 380–382.

[B11] Chen P. Li Z. Hu Y. (2016). Prevalence of Osteoporosis in China: a Meta-Analysis and Systematic Review. BMC Public Health 16 (1), 1039. 10.1186/s12889-016-3712-7 27716144 PMC5048652

[B12] Cheng C.-w. Wu T.-x. Shang H.-c. Li Y.-p. Altman D. G. Moher D. C.-C.F. Group (2017). CONSORT Extension for Chinese Herbal Medicine Formulas 2017: Recommendations, Explanation, and Elaboration (Traditional Chinese Version). Ann. Intern. Med. 167 (2), W7–W20. 10.7326/istranslatedfrom_m17-2977_1 28654988

[B13] Cosman F. de Beur S. J. LeBoff M. S. Lewiecki E. M. Tanner B. Randall S. (2014). Clinician's Guide to Prevention and Treatment of Osteoporosis. Osteoporos. Int. 25 (10), 2359–2381. 10.1007/s00198-014-2794-2 25182228 PMC4176573

[B14] Dang X. (2019). Clinical Study on Xianling Gubao Capsule Combined with Calci D600 in the Treatment of Osteoporosis. J. New Chin. Med. 51 (10), 162–164.

[B15] Ettinger M. P. (2003). Aging Bone and Osteoporosis. Arch. Intern. Med. 163 (18), 2237–2246. 10.1001/archinte.163.18.2237 14557222

[B16] Fang F. (2014). Clinical Observation of Xianling Gubao Combined with Alendronate in the Treatment of Primary Osteoporosis. Asia-Pacific Traditional Med. 10 (5), 105–106.

[B17] Fang X. M. Wang P. H. Yang Q. (2001). Observation of Therapeutic Effect of Xianling Gubao on Senile Osteoporosis. Mod. Rehabil. 5 (3), 87.

[B18] Feng Y. Huang Z. H. Qu R. L. (2018). Analysis of the Therapeutic Effect of Alendronic Acid Combined with Xianling Gubao in the Treatment of Primary Osteoporosis. Guangdong Med. J. 39 (15), 2385–2387.

[B19] Fu F. Y. Sun J. G. Wang R. T. Ye H. L. Tan B. Yan Y. (2020). Meta Analysis of the Curative Effect of Chinese Herbal Compound in Senile Osteoporosis. J. Hainan Med. Univ. 26 (2), 137–146.

[B20] Garnero P. (2008). Biomarkers for Osteoporosis Management. Mol. Diag Ther. 12 (3), 157–170. 10.1007/bf03256280 18510379

[B21] Gong J. C. Chen S. H. Xu W. G. Lu G. Q. (2012). Clinical Study on Xianling Gubao Combined with Alendronate in the Treatment of Osteoporosis with Deficiency of Kidney Yang. J. Traditional Chin. Orthopedics Traumatol. 24 (5), 327–329.

[B22] Guyatt G. H. Oxman A. D. Sultan S. Glasziou P. Akl E. A. Alonso-Coello P. (2011). GRADE Guidelines: 9. Rating up the Quality of Evidence. J. Clin. Epidemiol. 64 (12), 1311–1316. 10.1016/j.jclinepi.2011.06.004 21802902

[B23] Han C. T. (2016). A Comparative Study on the Curative Effect of Xianling Gubao Capsule Combined with Caltrate D and Alendronate Sodium Tablets in the Treatment of Senile Osteoporosis. Chin. Med. Mod. Distance Educ. China 14 (13), 92–93.

[B24] Hou X. S. Jiang W. X. Zhuang J. (2016). Clinical Observation of Xianling Gubao Combined with Sodium Alendate in the Treatment of Osteoporosis. China Pharm. 27 (17), 2391–2393.

[B25] Ishtiaq S. Fogelman I. Hampson G. (2015). Treatment of post-menopausal Osteoporosis: beyond Bisphosphonates. J. Endocrinol. Invest. 38 (1), 13–29. 10.1007/s40618-014-0152-z 25194424

[B26] Higgins J. Green S. (2011).in Cochrane Handbook for Systematic Reviews of Interventions Version 5.1.0 (Berlin: The Cochrane Collaboration), S38. Naunyn-Schmiedebergs Archiv für experimentelle Pathologie und Pharmakologie, 5.

[B27] Jiang Z. C. (2006). Clinical Observation of Xianling Gubao Capsule in Treating 30 Cases of Avascular Necrosis of Femoral Head. Chin. J. Traditional Med. Traumatol. Orthopedics 14, 56–57.

[B28] Jing Z. Wang C. Yang Q. Wei X. Jin Y. Meng Q. (2019). Luteolin Attenuates Glucocorticoid-Induced Osteoporosis by Regulating ERK/Lrp-5/GSK-3β Signaling Pathway *In Vivo* and *In Vitro* . J. Cel Physiol. 234 (4), 4472–4490. 10.1002/jcp.27252 30192012

[B29] Kang L. C. Li W. B. Lan H. Deng W. M. (2020). Effect of Xianling Gubao Capsule on Bone Mineral Density and Bone Metabolism in Senile Osteoporosis. Med. Innovation China 17 (22), 37–40.

[B30] Kanis J. A. Melton L. J. 3rd Christiansen C. Johnston C. C. Khaltaev N. (1994). The Diagnosis of Osteoporosis. J. Bone Miner Res. 9 (8), 1137–1141. 10.1002/jbmr.5650090802 7976495

[B31] Lewiecki E. M. (2010). Bisphosphonates for the Treatment of Osteoporosis: Insights for Clinicians. Ther. Adv. Chronic Dis. 1 (3), 115–128. 10.1177/2040622310374783 23251734 PMC3513863

[B32] Li A. (2018). Feasibility Research of Xianlinggubao Capsule in the Treatment of Female Climacteric Osteoporosis. Chin. Community Doctors 34 (18), 100–101.

[B33] Li D. P. Li S. J. Shan Y. Y. Zhou D. M. Tian L. (2018). Effect of Xianling Gubao Capsule on Bone Mineral Density and Bone Metabolism in Treatment of Patients with Osteoporosis Pain. Prog. Mod. Biomed. 18 (24), 4756–4759.

[B34] Li J. Sun K. Qi B. Feng G. Wang W. Sun. Q. (2020). An Evaluation of the Effects and Safety of Zuogui Pill for Treating Osteoporosis: Current Evidence for an Ancient Chinese Herbal Formula. Phytother Res. 35. 10.1002/ptr.6908 PMC8246738 33089589

[B35] Li J. Y. Jia Y. S. Chai L. M. Mu X. H. Ma S. Xu L. (2017). Effects of Chinese Herbal Formula Erxian Decoction for Treating Osteoporosis: a Systematic Review. Clin. Interv. Aging 12, 45–53. 10.2147/CIA.S117597 28115834 PMC5221555

[B36] Li M. Ni J. L. Hu M. Qiu X. P. (2019). Comparison of the Efficacy of Xianling Gubao Capsule Combined with Calcium D 600 and Alendronate Tablet in the Treatment of Senile Osteoporosis. Shanxi Med. J. 48 (3), 284–287.

[B37] Li Y. Q. (2020). Effect of Xianling Gubao Capsule Combined with Calcium Acetate Capsule on Osteoporosis in Postmenopausal Women. World J. Complex Med. 6 (6), 183–185.

[B38] Lin J. T. Lane J. M. (2003). Bisphosphonates. J. Am. Acad. Orthopaedic Surgeons 11 (1), 1–4. 10.5435/00124635-200301000-00001 12699366

[B39] Lin J. Zhu J. Wang Y. Zhang N. Gober H.-J. Qiu X. (2017). Chinese Single Herbs and Active Ingredients for Postmenopausal Osteoporosis: From Preclinical Evidence to Action Mechanism. Bst 11 (5), 496–506. 10.5582/bst.2017.01216 29151553

[B40] Liu B. J. (2014). Efficacy of Xianling Gubao Capsule Combined with Alendronate Sodium in the Treatment of Primary Osteoporosis. J. China Prescription Drug 12 (8), 54–55.

[B41] Liu B. Y. Bai R. (2016). Clinical Study of Xianling Gubao Capsule on Middle-Aged and Elderly Patients with Osteoporosis. Shaanxi J. Traditional Chin. Med. 37 (10), 1364–1365.

[B42] Liu Q. Chen D. Ye Z. Jin Z. Ma T. Huang X. (2020). Minodronate in the Treatment of Osteoporosis: A Systematic Review and Meta-Analysis. Medicine (Baltimore) 99 (40), e22542. 10.1097/md.0000000000022542 33019463 PMC7535701

[B43] Liu S., X. (2018). Study on the Effect and Safety of Xianling Gubao Combined with Alendronate in the Treatment of Osteoporosis. J. Pract. Gynecol. Endocrinol. 5 (13), 183–184.

[B44] Liu Y. He Y. (2011). Study on the Correlation between Blood Calcium Concentration and Osteoporosis. Mod. Med. J. 39 (2), 216–217.

[B45] Luo Y. Zheng S. Ding Y. Dai Y. Zhou Y. Xiang R. (2017). Erratum: Preventive Effects of Kudzu Root on Bone Loss and Cartilage Degradation in Ovariectomized Rats. Am. J. Transl Res. 9 (7), 5180–3527. 29218115 PMC5714801

[B46] Lv F. R. (2020). Therapeutic Effect of Tripeptide Combined with Xianling Gubao on Osteoporosis and its Influence on Bone Mineral Density. Smart Healthc. 6 (8), 195–196.

[B47] Ma F. Y. (2018). Xieling Gubao Capsule Combined with Methotrexate in the Treatment of 37 Cases of Rheumatoid Arthritis Secondary Osteoporosis. West. J. Traditional Chin. Med. 31 (2), 98–100.

[B48] Madore G. R. Sherman P. J. Lane J. M. (2004). Parathyroid Hormone. J. Am. Acad. Orthopaedic Surgeons 12 (2), 67–71. 10.5435/00124635-200403000-00001 15089079

[B49] Moher D. Liberati A. Tetzlaff J. Altman D. G. P. Group (2009). Preferred Reporting Items for Systematic Reviews and Meta-Analyses: the PRISMA Statement. Plos Med. 6 (7), e1000097. 10.1371/journal.pmed.1000097 19621072 PMC2707599

[B50] Ni J. Xiao J. D. (2019). Clinical Study of Xianling Gubao Capsule Combined with Calcium Carbonate D3 in the Treatment of Osteoporosis. J. China Prescription Drug 17 (7).

[B51] Ni L. G. Wang W. Li C. W. (2011). Current Research Status of Xianlinggubao for the Treatment of Osteoporotic Hip Fracture. Chin. J. Osteoporos., 1014–1018.*17*11.

[B52] Okada Y. Nawata M. Nakayamada S. Saito K. Tanaka Y. (2008). Alendronate Protects Premenopausal Women from Bone Loss and Fracture Associated with High-Dose Glucocorticoid Therapy. J. Rheumatol. 35 (11), 2249–2254. 10.3899/jrheum.080168 19031508

[B53] Orive M. Aguirre U. García-Gutiérrez S. Las Hayas C. Bilbao A. González N. (2015). Changes in Health-Related Quality of Life and Activities of Daily Living after Hip Fracture Because of a Fall in Elderly Patients: a Prospective Cohort Study. Int. J. Clin. Pract. 69 (4), 491–500. 10.1111/ijcp.12527 25721490

[B54] Osteoporosis and Bone Mineral Disease Branch of Chinese Medical Association (2017). Guidelines for the Diagnosis and Treatment of Primary Osteoporosis (2017). Chin. Gen. Pract. 20 (32), 3963–3982.

[B55] Osteoporosis Branch of Chinese Society of Gerontology and Gerontology (2019). Guidelines for the Diagnosis and Treatment of Senile Osteoporosis in China (2018). Chin. J. Gerontol. 39, 1–19.

[B56] Qin Y. Qiu B. Zhu &S. G. (2015). Analysis of the Curative Effect of Xianling Gubao Capsule on Osteoporosis and its Influence on Bone Metabolism and Bone Turnover index. Chin. J. Osteoporos. 12 (9), 1056–1060.

[B57] Rachner T. D. Khosla S. Hofbauer L. C. (2011). Osteoporosis: Now and the Future. The Lancet 377 (9773), 1276–1287. 10.1016/s0140-6736(10)62349-5 PMC3555696 21450337

[B58] Rogers M. (2003). New Insights into the Molecular Mechanisms of Action of Bisphosphonates. Cpd 9 (32), 2643–2658. 10.2174/1381612033453640 14529538

[B59] Sawka A. M. Papaioannou A. Adachi J. D. Gafni A. Hanley D. A. Thabane &L. (2005). Does Alendronate Reduce the Risk of Fracture in Men? A Meta-Analysis Incorporating Prior Knowledge of Anti-fracture Efficacy in Women. BMC Musculoskelet. Disord. 6, 39. 10.1186/1471-2474-6-39 16008835 PMC1182376

[B60] Shi Z. Y. Zhang X. G. Li C. W. Liu K. Liang B. C. Shi &X. L. (2017). Effect of Traditional Chinese Medicine Product, QiangGuYin, on Bone Mineral Density and Bone Turnover in Chinese Postmenopausal Osteoporosis. Evid. Based Complement. Alternat Med. 2017, 6062707. 10.1155/2017/6062707 28512501 PMC5415859

[B61] Si L. Winzenberg T. M. Jiang Q. Chen M. Palmer A. J. (2015). Projection of Osteoporosis-Related Fractures and Costs in China: 2010-2050. Osteoporos. Int. 26 (7), 1929–1937. 10.1007/s00198-015-3093-2 25761729

[B62] Society T. O. C. o. C. G. (2000). Chinese Osteoporosis Recommended Diagnostic Criteria (Second Draft). Chin. J. Osteoporos. 6 (1), 1–3.

[B63] Tan Y. Qiu B. Zhu S. G. Luo C. Chen L. Song H. (2015). Analysis of the Efficacy of Xianlinggubao Capsule on the Tre Atment of Oste Oporosis and its Influence S in the Marke Rs of Bone Metabolism and Bone Turnove R. Chin. J. Osteoporos. 21 (9).

[B64] Tu K. N. Lie J. D. Wan C. K. V. Cameron M. Austel A. G. Nguyen J. K. (2018). Osteoporosis: A Review of Treatment Options. P T 43 (2), 92–104. 29386866 PMC5768298

[B65] Wang F. L. Yang S. R. Li S. J. Sun C. Z. (2020a). Effects of Xianling Gubao Capsules Combined with Alendronate on Bone Metabolism and Bone Turnover Indexes in Patients with Osteoporosis. Drug Eval. Res. 43 (11), 2259–2262.

[B66] Wang F. Shi L. Zhang Y. Wang K. Pei F. Zhu H. (2018). A Traditional Herbal Formula Xianlinggubao for Pain Control and Function Improvement in Patients with Knee and Hand Osteoarthritis: A Multicenter, Randomized, Open-Label, Controlled Trial. Evid. Based Complement. Alternat Med. 2018, 1827528. 10.1155/2018/1827528 29619064 PMC5829359

[B67] Wang S. S. Wang J. P. (2017). Evaluation of Clinical Efficacy of Xianling Gubao Capsule Combined with salmon Calcitonin and Alendronate in the Treatment of Osteoporosis. Chin. Prim. Health Care 31 (4), 65–66.

[B68] Wang X. He Y. Guo B. Tsang M. C. Tu F. Dai Y. (2015). *In Vivo* screening for Anti-osteoporotic Fraction from Extract of Herbal Formula Xianlinggubao in Ovariectomized Mice. PLoS One 10 (2), e0118184. 10.1371/journal.pone.0118184 25695519 PMC4335011

[B69] Wang Y. Guo W. S. Cheng L. M. Liu P. Xia Z. Q. Wang H. D. (2020b). The Effect of Liuwei Dihuang Pill on the Treatment of Postmenopausal Osteoporosis: A Systematic Review and Meta-Analysis. Chin. J. Osteoporos. 26 (5), 663–671.

[B70] Wei S. (2013). Clinical Observation on 98 Cases of Primary Osteoporosis Treated by Xianling Gubao Capsule Combined with Sodium Alendronate. Asia-Pacific Traditional Med. 9 (1), 173–174.

[B71] Wright N. C. Looker A. C. Saag K. G. Curtis J. R. Delzell E. S. Randall S. (2014). The Recent Prevalence of Osteoporosis and Low Bone Mass in the United States Based on Bone mineral Density at the Femoral Neck or Lumbar Spine. J. Bone Miner Res. 29 (11), 2520–2526. 10.1002/jbmr.2269 24771492 PMC4757905

[B72] Wright N. C. Saag K. G. Dawson-Hughes B. Khosla S. Siris E. S. (2017). The Impact of the New National Bone Health Alliance (NBHA) Diagnostic Criteria on the Prevalence of Osteoporosis in the USA. Osteoporos. Int. 28 (4), 1225–1232. 10.1007/s00198-016-3865-3 27966104

[B73] Xie Y. M. Yu Y. Y. Dong F. H. Song S. C. Wang H. M. Liu Q. S. (2012). Clinical Practice Guideline of Traditional Chinese Medicine for Primary Osteoporosis(extract). China J. Traditional Chin. Med. Pharm. 27 (7), 1886–1890.

[B74] Xing Y. Bi H. Y. Zhang Q. N. (2013). Introduction of Common Chinese Patent Medicines for the Treatment of Osteoporosis. Chin. J. Osteoporos. 19 (1), 83–85.

[B75] Xu J. J. Yu J. H. Ying Y. M. Tong S. L. (2015). Clinical Observation of Xianling Gubao Combined with Alendronate in the Treatment of Osteoporosis. J. New Chin. Med. 47 (5), 148–149.

[B76] Xu M. Liu B. X. Huang C. J. Tang F. Y. Lou Y. M. Liang Z. (2009). Clinical Observation of Xianling Gubao Combined with Alendronate Sodium in the Treatment of Postmenopausal Osteoporosis. J. Liaoning Univ. Traditional Chin. Med. 11 (1), 94–95.

[B77] Xu Z. Chen H. W. Ren H. F. Jiang X. B. (2020). Clinical Study of Xianling Gubao Capsule Combined with Osteopeptide for Injection in the Treatment of Osteoporosis. Chin. Foreign Med. Res. 18 (3), 51–53.

[B78] Yan N. (2018). Effect of Xianling Gubao Capsule on the Cytokines, Bone Metabolism and Bone Turnover Indicators in Patients with Osteoporosis. J. Changchun Univ. Chin. Med. 34 (2), 299–302.

[B79] Ye F. Lan S. H. Huang S. M. Ye J. F. (2018). The Effect of Xianling Gubao Capsule Combined with Alendronate Sodium Tablets on Osteoporosis and the Effect of Serum Bone Metabolism, Inflammatory Factors and Oxidative Stress Levels. Chin. Arch. Traditional Chin. Med. 36 (11), 2709–2712.

[B80] Yu Y. B. (2014). Clinical Observation of Xianling Gubao Combined with Alendronate Sodium in the Treatment of Primary Osteoporosis. Chin. Med. Mod. Distance Educ. China 12 (20), 62–63.

[B81] Yuan Z. Min J. Zhao Y. Cheng Q. Wang K. Lin S. (2018). Quercetin Rescued TNF-Alpha-Induced Impairments in Bone Marrow-Derived Mesenchymal Stem Cell Osteogenesis and Improved Osteoporosis in Rats. Am. J. Transl Res. 10 (12), 4313–4321. 30662673 PMC6325508

[B82] Zhang G. Qin L. Shi Y. (2007). Epimedium-derived Phytoestrogen Flavonoids Exert Beneficial Effect on Preventing Bone Loss in Late Postmenopausal Women: a 24-month Randomized, Double-Blind and Placebo-Controlled Trial. J. Bone Miner Res. 22 (7), 1072–1079. 10.1359/jbmr.070405 17419678

[B83] Zhang M. Ying C. Li M. Gao S. S. Su &K. Z. (2020). Clinical Study on Xianling Gubao Capsule Combined with Alendronate Sodium Tablets in Treatment of Senile Osteoporosis. J. New Chin. Med. 52 (21), 34–37.

[B84] Zhang N.-D. Han T. Huang B.-K. Rahman K. Jiang Y.-P. Xu H.-T. (2016). Traditional Chinese Medicine Formulas for the Treatment of Osteoporosis: Implication for Antiosteoporotic Drug Discovery. J. Ethnopharmacology 189, 61–80. 10.1016/j.jep.2016.05.025 27180315

[B85] Zhang P. (2017). Study on the Curative Effect and Safety of Xianlinggubao Combined with Alendronate in the Treatment of Osteoporosis. Chin. Community Doctors 33 (12), 81–84.

[B86] Zhang Z. Song C. Fu X. Liu M. Li Y. Pan J. (2014). High-dose Diosgenin Reduces Bone Loss in Ovariectomized Rats via Attenuation of the RANKL/OPG Ratio. Ijms 15 (9), 17130–17147. 10.3390/ijms150917130 25257532 PMC4200779

[B87] Zhao J. Wu J. Xu B. Yuan Z. Leng Y. Min J. (2019). Kaempferol Promotes Bone Formation in Part via the mTOR Signaling Pathway. Mol. Med. Rep. 20 (6), 5197–5207. 10.3892/mmr.2019.10747 31638215 PMC6854588

[B88] Zheng Z.-G. Zhang X. Zhou Y.-P. Lu C. Thu P. M. Qian C. (2017). Anhydroicaritin, a SREBPs Inhibitor, Inhibits RANKL-Induced Osteoclastic Differentiation and Improves Diabetic Osteoporosis in STZ-Induced Mice. Eur. J. Pharmacol. 809, 156–162. 10.1016/j.ejphar.2017.05.017 28501578

[B89] Zhou J. H. (2020). Effects of Xianling Gubao Combined with Alendronate Sodium on Bone Metabolism Indexes, Bone Density and Bone Pain Symptom in Postmenopausal Osteoporosis. Chin. J. Gerontol. 40 (3), 581–584.

[B90] Zhou Y. S. Qiu L. Ye H. T. He Z. M. (2016). Analysis of Clinical Efficacy of Xianling Gubao Combined with Alendronate in the Treatment of Osteoporosis. Drugs and Clinic 13 (13), 21–27.

[B91] Zhu H. M. Qin L. Garnero P. Genant H. K. Zhang G. Dai K. (2012). The First Multicenter and Randomized Clinical Trial of Herbal Fufang for Treatment of Postmenopausal Osteoporosis. Osteoporos. Int. 23 (4), 1317–1327. 10.1007/s00198-011-1577-2 21505910

[B92] Zhuang L. F. (2013). Clinical Observation of Xianling Gubao Combined with Alendronate in the Treatment of Postmenopausal Osteoporosis. Zhejiang J. Integrated Traditional Chin. West. Med. 23 (7), 558–560.

